# Determinants of Knowledge About Dietary Supplements Among Polish Internet Users: Nationwide Cross-sectional Study

**DOI:** 10.2196/25228

**Published:** 2021-04-21

**Authors:** Michał Seweryn Karbownik, Robert Horne, Ewelina Paul, Edward Kowalczyk, Janusz Szemraj

**Affiliations:** 1 Department of Pharmacology and Toxicology Medical University of Lodz Lodz Poland; 2 Centre for Behavioural Medicine The School of Pharmacy University College London London United Kingdom; 3 Osom Studio Lodz Poland; 4 Department of Medical Biochemistry Medical University of Lodz Lodz Poland

**Keywords:** dietary supplements, knowledge, beliefs, advertising, education, statistical model, health services, Poland, online social networking

## Abstract

**Background:**

An accurate understanding of dietary supplements (DS) is a prerequisite for informed decisions regarding their intake. However, there is a need for studies on this understanding among the public based on validated research tools.

**Objective:**

This study aims to assess the knowledge about DS among Polish internet users with no medical education and to identify its determinants and design an appropriate predictive model.

**Methods:**

The study protocol was prospectively registered with a statistical analysis plan. Polish users of a web-based health service and a social networking service were administered a survey consisting of the recently developed questionnaire on knowledge about DS, the questionnaire on trust in advertising DS, the beliefs about medicines questionnaire, and several other health-related single-item measures and sociodemographic questions. The results were subjected to general linear modeling.

**Results:**

A total of 6273 participants were included. Of the 17 yes or no questions in the questionnaire of knowledge about DS, the mean number of correct responses was 9.0 (95% CI 8.9-9.1). Health service users performed worse than social networking users by 2.3 points (95% CI 2.1-2.5) in an analysis adjusted for potential confounders. Internet users had fewer true beliefs about DS if they presented higher trust in their advertising (adjusted β=−.37; 95% CI −.39 to −.34), used DS (adjusted β=−.14; 95% CI −.17 to −.12), experienced their positive effect (adjusted β=−.16; 95% CI −.18 to −.13), were older or younger than 35 years (adjusted β=−.14; 95% CI −.17 to −.12), expressed interest in the topic of DS (adjusted β=−.10; 95% CI −.13 to −.08), reported getting information about the products from friends (adjusted β=−.13; 95% CI −.15 to −.11), and believed that medicines are harmful (adjusted β=−.12; 95% CI −.15 to −.10). The proposed 5-predictor model could explain 31.2% of the variance in knowledge about DS. The model appeared resistant to overfitting and was able to forecast most of the observed associations.

**Conclusions:**

Polish internet users with no medical education exhibit some false beliefs regarding DS. Trusting the advertising of DS appears to conflict with knowledge about them. There is an urgent need for effective web-based educational campaigns on DS and the promotion of advertising literacy. After the proposed predictive model is externally validated, it may help identify the least informed target audience.

## Introduction

### Background

Dietary supplements (DS) are consumed by approximately half of the adult population in developed countries [1-3]. They are used not only to fill potential nutrient gaps, as they are intended to, but also ostensibly to improve and maintain overall health and well-being, prevent or treat diseases, enhance cognitive and sports performance, and extend life expectancy [3-8]. The recommendations of health care providers seem to play only a minor role in the decision to use DS [4]. Instead, media, including the internet, appear to shape DS consumption patterns [5,9]. The internet is able to build a highly positive picture of DS [10-15]. In contrast, recent findings of large-scale, high-quality research studies highlight overall negligible benefits [2,16] and potential threats [2,17,18] related to DS use. It is important to evaluate the actual knowledge about DS held by the public and its determinants, not only for cognitive reasons but also for tailoring educational campaigns capable of fostering informed decisions regarding DS consumption [3,19-22].

Knowledge about DS has already been examined in numerous research studies worldwide, in multiple populations [3,23-29], including the general public [30-33]. Most participants were likely internet users [34]. Although the findings consistently reported an inadequate knowledge level, a systematic review could not draw any conclusions regarding knowledge about DS because of the heterogeneity of the data [35]. Moreover, most of the reports had multiple methodological limitations: the sample sizes were fairly low and selected with highly nonprobabilistic techniques, and the methods applied to examine knowledge about DS presented modest or vaguely described validity proofs. Apart from a general nutrition knowledge questionnaire [36] and similar measures for health care workers [28,37], no satisfactorily validated tool was available to test knowledge about DS in the general population until such a test was developed in 2019 [38]. The questionnaire exhibited acceptable and well-documented validity and was designed to screen commonly identifiable beliefs that are important from the public health perspective. This questionnaire was developed in the same country and the same language as this research was performed, thus allowing a valid assessment of knowledge about DS and its determinants on a large scale.

### Objectives

This study aims to evaluate the level of knowledge about DS and identify its determinants among adult Polish internet users without medical education. The following research questions were addressed: (1) what is the level of knowledge about DS among Polish internet users? (2) what are the characteristics of the population members who are unknowledgeable about DS? and (3) how can the level of knowledge about DS be modeled in this population? These research questions were additionally addressed to some subpopulations with a high impact on public health: older people, residents of rural areas, and people with low earnings.

## Methods

### Ethical Considerations

The study was approved by the Bioethics Committee of the Medical University of Lodz, Poland (KE/1382/19, received on October 15, 2019). Expressing informed consent in an electronic manner was mandatory to participate in the study. The study protocol with the data analysis plan was prospectively registered on November 26, 2019, in the public repository Open Science Framework (Center for Open Science; Open Science Framework) [39]. A few changes to the preregistered study protocol were made after the study commencement, which are presented and discussed in Multimedia Appendix 1. DOZ.pl (Pelion), a web-based health service entity, was the research partner in this study. The only role of DOZ.pl was to prepare promotional materials (each one approved by the researchers) to help recruit participants and to enter the survey content (authored by the researchers with no influence of DOZ.pl) into an external web-based survey system (see section *Research Instruments*). This paper was outlined according to the STROBE (Strengthening the Reporting of Observational Studies in Epidemiology) guidelines for cross-sectional studies [40] and, to some extent, the TRIPOD (Transparent Reporting of a multivariable prediction model for Individual Prognosis Or Diagnosis) guidelines [41].

### Study Design

This was a nationwide cross-sectional study conducted among Polish internet users. A self-administered survey was accessed on the web. Each participant was asked to complete the survey once.

### Research Instruments

The survey was created using the Survio web-based survey system (Survio). It was pretested in a qualitative manner by 5 nonmedically educated people (3 women; mean age 52.0 years, SD 19.1 years) for readability, understanding, appearance, face validity, and time to survey completion according to the procedure described in a study by Hilton [42]. The survey was modified according to the feedback received to construct the final version.

The survey comprised 5 consecutive parts:

1. *Introduction*: the first page provided the volunteers with basic information about the purpose of the study, the structure of the survey, the rights of participants, and contact person data. This page also included a statement of informed consent, which was to be expressed electronically by ticking a Start the survey now button.
2. *Knowledge about DS*: this part included a recently developed and validated questionnaire on knowledge about DS in its original Polish language version [38]. The questionnaire consisted of 17 true-or-false statements; the respondent received a point for each correct answer with a maximum of 17 points. The questionnaire was formed of 2 subscales to examine general and specific knowledge. Knowledge about DS–general assessed “familiarity with the useful facts concerning the legal status of DS in general” [38]. It included 7 items related to DS definition, DS quality standards, and DS package labeling. Knowledge about DS–specific assessed “familiarity with common, scientifically proven and useful facts about popular dietary supplements” [38]. It included 10 items related to the efficacy of vitamin C, vitamin D, multivitamin, calcium, magnesium, and antioxidants and their adverse effects and oral absorption. The content of each questionnaire item is presented in Multimedia Appendix 2. The sum of correct answers in knowledge about DS–general and knowledge about DS–specific formed knowledge about DS–total, which operationalized the construct of knowledge about DS.
3. *Dietary supplement advertising*: this part included a single-item measure of having contact with DS advertisements within the past week and a recently developed and validated questionnaire on trust in advertising DS in its original Polish version [38]. The tool consisted of 8 pairs of opposing expressions characterizing DS advertisements and information conveyed by them to be assessed on a 5-point semantic differential scale. The questionnaire was formed from the reliability, intelligibility, and affect subscales. The sum of scores on the subscales operationalized the construct of the trust in advertising DS.
4. *Beliefs about medicines*: this part included the Polish version [43] of the beliefs about medicines questionnaire (BMQ) [44]. The tool included in this study involved only the BMQ-General, as it may be used separately from BMQ-Specific to assess ideas about medicines in general among people who may take no medicines [44]. The BMQ-General consisted of 8 statements for a respondent to express their opinions using a 5-point Likert scale. The BMQ-General was composed of 2 separate 4-item subscales to operationalize the construct of beliefs that medicines are overused by doctors (BMQ Overuse) and the construct of beliefs that medicines are harmful, addictive, poisonous, and should not be taken continuously (BMQ Harm).
5. *Other medical and sociodemographic data*: this part included a set of single-item measures of health (4-point Likert scale), diet and physical activity (5-point semantic differential scale), use of any DS within the past 30 days (further called use of DS; 2-point Likert scale), personal experience of positive (or negative) effect of DS (further called positive [or negative] effect of DS, for DS users only; both 2-point Likert scale), interest in DS (5-point semantic differential scale), sources of getting knowledge about DS (assessed in 5 categories: medical doctors, pharmacists, dieticians, friends with no medical education, and media such as magazines, television, radio, and internet; each of the categories assessed in 4-point Likert scale), and conventional cigarette smoking and electronic cigarette use (both as 3-point Likert scales: never; no, but I smoked/used in the past; and yes). A measure of self-rated diet used in this study was found in a pretest study (a convenience sample of 117 healthy adults from the general population and medical students) to significantly correlate (Pearson *r*=0.48; *P*<.001) with the Polish version (own translation with no full validation) of the Starting the Conversation scale, a brief dietary assessment tool [45]. In the same pretest study, a measure of self-rated physical activity was found to significantly correlate with the Polish version of the International Physical Activity Questionnaire Short Form [46] (Pearson *r*=0.49; *P*<.001). Demographic data in the survey used in this study included age, sex, educational level (with 5 options to choose from), having medical education (*no* or *yes*), number of inhabitants in a place of residence, and monthly net household earnings per family member (both with 4 options to choose from).

None of the survey questions employed forced answering. In all semantic differential scales, the central value was set as the default answer. It was predicted that the survey would take 5 to 10 minutes to complete. After completing the survey, participants were provided with correct answers to the knowledge about DS questionnaire with expert comments for educational purposes. A survey could be completed only once from a single internet protocol address to avoid duplicate records.

The detailed characteristics of the survey questions and their method of operationalization are described in the study protocol [39]. The questions asked in the survey and its layout are presented in Polish (original version) and English in Multimedia Appendix 3.

### Participants

The desired sample size was set to 10,000 participants. It was determined not according to the analysis of statistical power but based on the estimated ability of DOZ.pl to reach the audience. A nonprobability convenience sampling technique was used to recruit internet users. They were accessed through a web-based health service and a general social networking website between November 26, 2019, and March 11, 2020. DOZ.pl was used as the health service, whereas Wykop.pl (Wykop; modeled after the American Digg service) was the social networking service. In the 3 months from December 2019 to February 2020, when the study was conducted, DOZ.pl was the sixth most popular web-based health service and the first most popular web-based pharmacy in Poland, with a mean of 37.91 million page views and 4.39 million unique users (15.72% of internet users in Poland) per month. In the same period, Wykop.pl was the fifth-to-sixth most popular web-based social networking service in Poland, with 74.31 million page views and 4.28 million unique users (15.36% of internet users in Poland) each month [47].

Apart from the refusal of electronic informed consent, there were no specific exclusion criteria in the study. The survey could be completed by anybody who reached it on the web; however, a knowledge of Polish was needed to complete it.

### Procedure

Throughout the period during which the study was conducted, promotional material encouraging participation with a link to the survey was temporarily placed in a slider on the main DOZ.pl website and some subpages, DOZ.pl social media (Facebook), and some other DOZ.pl channels. Moreover, the invitation to participate was emailed twice to the subscribers of DOZ.pl newsletter. Similarly, the promotional material with a link to the survey was posted on the Wykop.pl website to be entered and promoted by service users.

### Data Analysis

Each survey record was assumed to be completed by a single respondent. Before performing the analysis, survey data were cleaned by removing records considered potentially meaningless [48] (as detailed in Multimedia Appendix 4). The number, frequency, and pattern of missing values were examined (Multimedia Appendix 5). Before any further analysis, the missing values were completed using a multiple imputation by chained equation procedure under a *missing at random* assumption about the unobserved data.

Details of the data analysis are depicted in a preregistered data analysis plan [39]. Briefly, descriptive statistics were presented, and participants’ characteristics from the 2 web-based services were compared using the asymptotic Mann-Whitney *U* test, Pearson chi-square test, and general linear models (GLMs). Then, the associations between all the examined characteristics of participants and knowledge about DS–general, knowledge about DS–specific, and knowledge about DS–total were tested. The associations were examined in raw analyses and with adjustment for potential confounders (age, following its transformation; sex; education; the number of inhabitants; earnings; type of web service through which the survey was accessed; and calendar year in which a participant completed the survey). Adjustment for calendar year was performed to correct for the potential effect of “Broadcasting agreement about the rules and regulations for advertising dietary supplements,” which was signed in Poland and became effective on January 01, 2020. Although the Likert and semantic differential scale data should be perceived as ordinal variables, parametric tests were used to allow for multivariate modeling with GLM. Sensitivity analysis of the associations was performed in 2 steps. First, univariate associations were tested using a corresponding nonparametric procedure (Spearman rho). Second, all parametric associations were repeated in a complete case database before data imputation. The Benjamini and Hochberg procedure was used to reduce the false discovery rate to 0.05, which was inflated by testing multiple hypotheses.

The predictive model of knowledge about DS was built using multivariate linear regression analysis. The selection of knowledge about DS predictors was based on the following criteria: first, the characteristics substantially associated with the knowledge about DS–total were preferred. Second, objective measures, which reflect the underlying constructs with strong proof of validity, were favored. Third, a set of predictors with negligible collinearity was retained. Collinearity was assessed using explanatory factor analysis and Pearson *r* correlation matrix. Data transformation was also considered. The final model was selected according to the best subset selection algorithm to promote model simplicity based on the Akaike and Bayesian information criteria. Its performance was illustrated with a calibration plot as well as mean absolute error (MAE) of prediction (the mean difference between predicted and observed knowledge about DS–total score) and root mean squared error (RMSE). The final model was internally validated using a 10-fold cross-validation procedure to correct for overfitting bias.

*P* lower than Benjamini-Hochberg corrected significance level or *P*<.05 were considered statistically significant. The analyses were performed using STATISTICA software version 13.3 (Statsoft) and R software version 4.0.0 (package *mice* version 3.8.0; R Foundation for Statistical Computing).

## Results

### Database

The survey was displayed 24,400 times and completed 7632 times (7632/24,400, 31.28% of the displayed surveys). A total of 6273 records (6273/7632, 82.19% of the completed records) were retained in the final database following the cleaning procedure, which is detailed in Multimedia Appendix 4. Missing data comprised 0.38% of the values in the database, and convincing evidence was found against the pattern of *missing completely at random*. Details of the data missingness analysis are presented in Multimedia Appendices 5 and 6. Participants completed the survey in a median time of 6 minutes and 25 seconds (first to third quartile: 5 minutes and 9 seconds to 8 minutes and 34 seconds).

### Study Participants Characteristics

Following the missing data imputation, out of 6273 study participants, 3640 (58.03%) were female. The study participants’ mean age was 38.4 years (SD 13.4 years; range 18-90 years). A total of 64.05% (4018/6273) of participants accessed the survey through the health service and 35.95% (2255/6273) through the social networking service.

Substantial differences were found between the participants from different web-based services. Those from the health service—according to their reports—were older, mostly women, living in a smaller place of residence, and a little better educated but earning less money. Although they reported having worse overall health, they tended to eat a healthier diet, engage in more physical activity, and were less likely to smoke cigarettes or use e-cigarettes. Health service visitors were more negative about medicines, more likely to use DS, and were more interested in DS issues. They also declared having more contact with DS advertisements and trusting them more. Media were reported to be the major source of knowledge about DS, irrespective of the type of web-based service used, followed by pharmacists, medical doctors, friends, and dieticians. Detailed characteristics of the study participants, with differences between the participants from 2 web-based services, are presented in Table 1.

**Table 1:** Sociodemographic characteristics, health-related characteristics, and dietary supplement–related characteristics of the study participants. Data for the total sample and the comparison of participant characteristics from different web-based services are provided.

Characteristics						Total sample (N=6273)			Differences between the type of web-based service															
									Health service users (n=4018)			Social networking service users (n=2255)			Test statistics and *P* values for the comparison^a^									
															*Z* value				*χ*^2^ (*df*)				*P* value	
**Sociodemographic**																								
	**Age**															32.86				N/A^b^				<.001
		Values (years), mean (SD)			38.4 (13.4)			42.6 (14.3)			31.1 (7.2)													
		Values (years), median (Q_1_-Q_3_)^c^			35 (28-46)			40 (31-53)			30 (26-35)													
	**Sex**															N/A				2305.1 (1)				*<*.001
		Female, n (%)			3640 (58.03)			3232 (80.44)			408 (18.09)													
		Male, n (%)			2633 (41.97)			786 (19.56)			1847 (81.91)													
	**Education**															3.05				N/A				.002
		Primary, n (%)			42 (0.67)			30 (0.75)			12 (0.53)													
		Secondary or vocational, n (%)			1874 (29.87)			1205 (29.99)			669 (29.67)													
		Bachelor, n (%)			1222 (19.48)			686 (17.07)			536 (23.77)													
		Master, n (%)			3000 (47.82)			1997 (49.70)			1003 (44.48)													
		Doctorate, n (%)			135 (2.15)			100 (2.49)			35 (1.55)													
	**Number of inhabitants**															−6.87				N/A				*<*.001
		Below 5000, n (%)			783 (12.48)			532 (13.24)			251 (11.13)													
		5000-50,000, n (%)			1250 (19.93)			865 (21.53)			385 (17.07)													
		50,000-500,000, n (%)			2105 (33.56)			1377 (34.27)			728 (32.28)													
		Over 500,000, n (%)			2135 (34.03)			1244 (30.96)			891 (39.51)													
	**Earnings, PLN^d^ (US $)**															−23.67				N/A				*<*.001
		Below 1000 (256), n (%)			369 (5.88)			297 (7.39)			72 (3.19)													
		1000-2000 (256-512), n (%)			1396 (22.25)			1139 (28.35)			257 (11.40)													
		2000-3000 (512-768), n (%)			1856 (29.59)			1320 (32.85)			536 (23.77)													
		Over 3000 (768), n (%)			2652 (42.28)			1262 (31.41)			1390 (61.64)													
**Health-related**																								
	**Overall health**																							
		**Health status**														−9.60				N/A				*<*.001
			Values, mean (SD)	2.59 (0.79)			2.52 (0.78)			2.71 (0.80)														
			Values, median (Q_1_-Q_3_)	3 (2-3)			3 (2-3)			3 (2-3)														
		**Diet**														17.73				N/A				*<*.001
			Values, mean (SD)	3.37 (0.92)			3.53 (0.85)			3.09 (0.98)														
			Values, median (Q_1_-Q_3_)	3 (3-4)			4 (3-4)			3 (2-4)														
		**Physical activity**														4.68				N/A				*<*.001
			Values, mean (SD)	2.76 (1.10)			2.81 (1.05)			2.68 (1.16)														
			Values, median (Q_1_-Q_3_)	3 (2-4)			3 (2-4)			3 (2-4)														
	**Nicotine status**																							
		Current cigarette smoker, n (%)			785 (12.51)			401 (9.98)			384 (17.03)			N/A				65.5 (1)				*<*.001		
		Past but not current cigarette smoker, n (%)			1251 (19.94)			746 (18.57)			505 (22.39)			N/A				13.3 (1)				*<*.001		
		Current e-cigarette user, n (%)			343 (5.47)			94 (2.34)			249 (11.04)			N/A				211.6 (1)				*<*.001		
		Past but not current e-cigarette user, n (%)			260 (4.14)			100 (2.49)			160 (7.10)			N/A				77.1 (1)				*<*.001		
	**Beliefs about medicines**																							
		**Overuse**														14.98				N/A				*<*.001
			Values, mean (SD)	12.9 (3.5)			13.4 (3.5)			12.0 (3.4)														
			Values, median (Q_1_-Q_3_)	13 (10-16)			14 (11-16)			12 (9-15)														
		**Harm**														15.28				N/A				*<*.001
			Values, mean (SD)	9.5 (3.3)			10.0 (3.4)			8.6 (8.6)														
			Values, median (Q_1_-Q_3_)	9 (7-12)			10 (7-12)			8 (6-11)														
**DS^e^-related**																								
	**Use**																							
			Use of DS^f^, n (%)	4615 (73.57)			3311 (82.40)			1304 (57.83)			N/A				448.7 (1)				*<*.001			
			Positive effect^g^ of DS, n (%)	3162 (68.52)			2330 (70.37)			832 (63.80)			N/A				18.7 (1)				*<*.001			
			Negative effect^g^ of DS, n (%)	169 (3.66)			126 (3.81)			43 (3.30)			N/A				0.7 (1)				.41			
	**Advertising**																							
			Having contact with DS advertisements^h^, n (%)	5418 (86.37)			3603 (89.67)			1815 (80.49)			N/A				103.5 (1)				*<*.001			
	**Trust in DS advertisements**															27.96				N/A				*<*.001
			Values, mean (SD)	17.7 (5.8)			19.2 (5.9)			15.0 (4.5)														
			Values, median (Q_1_-Q_3_)	17 (13-22)			19 (15-24)			15 (12-18)														
	**Interest in DS**															26.45				N/A				*<*.001
			Values, mean (SD)	2.9 (1.1)			3.2 (1.0)			2.4 (1.1)														
			Values, median (Q_1_-Q_3_)	3 (2-4)			3 (3-4)			2 (1-3)														
	**Getting knowledge from**																							
		**Medical doctors**														11.93				N/A				*<*.001
			Values, mean (SD)	0.72 (0.81)			0.80 (0.81)			0.57 (0.77)														
			Values, median (Q_1_-Q_3_)	1 (0-1)			1 (0-1)			0 (0-1)														
		**Pharmacists**														18.63				N/A				*<*.001
			Values, mean (SD)	0.88 (0.85)			1.02 (0.86)			0.62 (0.78)														
			Values, median (Q_1_-Q_3_)	1 (0-1)			1 (0-2)			0 (0-1)														
		**Dieticians**														6.77				N/A				*<*.001
			Values, mean (SD)	0.47 (0.78)			0.51 (0.81)			0.38 (0.73)														
			Values, median (Q_1_-Q_3_)	0 (0-1)			0 (0-1)			0 (0-1)														
		**Friends**														9.98				N/A				*<*.001
			Values, mean (SD)	0.68 (0.76)			0.75 (0.78)			0.55 (0.69)														
			Values, median (Q_1_-Q_3_)	1 (0-1)			1 (0-1)			0 (0-1)														
		**Media**														12.61				N/A				*<*.001
			Values, mean (SD)	1.41 (1.04)			1.53 (1.00)			1.19 (1.06)														
			Values, median (Q_1_-Q_3_)	1 (1-2)			2 (1-2)			1 (0-2)														

^a^Asysmptotic Mann-Whitney *U* test (*Z* statistic is provided) or chi-square test (χ^2^_df_ is provided); Benjamini-Hochberg corrected significance level: 0.048.

^b^N/A: not applicable.

^c^Q_1_-Q_3_: 1st to 3rd quartile.

^d^PLN: Polish złoty. PLN was converted to US $ according to the average exchange rate on the study beginning date (source: Narodowy Bank Polski).

^e^DS: dietary supplements.

^f^Within the past 30 days.

^g^Frequency calculated in relation to the number of dietary supplements users.

^h^Within the past week.

### Knowledge About DS

The knowledge about DS of web-based service users could be assessed as low, being not much better than a random guess, the success rate of which is 50% for binary questions. Knowledge about DS–general presented overall better results than knowledge about DS–specific. Knowledge about DS of health service users was lower than that of social networking users in both its subscales, approaching an effect size of 6%, as expressed by partial eta-squared, in an analysis adjusted for potential confounders. Details of the knowledge about DS analysis of web-based service users are presented in Table 2. The numbers and frequencies of correct answers to each item of the knowledge about DS questionnaire are presented in Multimedia Appendix 2.

**Table 2:** Knowledge about dietary supplements among web-based health service users and social networking service users.

Variable	Total sample (N=6273)		Differences between the type of web-based service							
			Type of analysis^a^	Health service users (n=4018), mean (95% CI)	Social networking service users (n=2255), mean (95% CI)	Effect size of the difference^b^		Test statistics and *P* values for the comparison		
	Mean	95% CI				pη^2^ value (%)^c^	Mean (95% CI)	*F* test (*df*)	*F* test (*df*)	*P* value
**Knowledge about dietary supplements–General^d^**	4.4	4.4-4.5								
			Raw	4.0 (3.9-4.1)	5.3 (5.2-5.3)	8.7	−1.3 (−1.4 to −1.2)	595.4 (1,6271)	N/A^e^	*<*.001
			Adjusted	4.1 (4.1-4.2)	5.0 (4.9-5.1)	2.0	−0.9 (−1.0 to −0.7)	N/A	129.7 (1,6265)	*<*.001
**Knowledge about dietary supplements–Specific^f^**	4.5	4.5-4.6								
			Raw	4.0 (3.9-4.0)	5.5 (5.4-5.6)	13.8	−1.5 (−1.6 to −1.4)	1008.1 (1,6271)	N/A	*<*.001
			Adjusted	4.0 (4.0-4.1)	5.5 (5.4-5.5)	6.2	−1.4 (−1.5 to −1.3)	N/A	411.9 (1,6265)	*<*.001
**Knowledge about dietary supplements–Total^g^**	9.0	8.9-9.1								
			Raw	8.0 (7.9-8.1)	10.8 (10.7-10.9)	16.5	−2.8 (−3.0 to −2.7)	1237.9 (1,6271)	N/A	*<*.001
			Adjusted	8.2 (8.1-8.3)	10.5 (10.3-10.6)	5.9	−2.3 (−2.5 to −2.1)	N/A	393.4 (1,6265)	*<*.001

^a^Raw analyses: performed only with the variables reported; adjusted analyses: adjusted for |Age−35|, sex, education, number of inhabitants, earnings, and calendar year—all included as linear factors; estimates in adjusted analyses reported as estimated marginal means.

^b^Reported as a partial eta-squared (pη^2^) and a difference between knowledge about dietary supplements of health service users and knowledge about dietary supplements of social networking service users (95% CI).

^c^pη^2^: partial eta-squared.

^d^An expected result of random guess is 3.5.

^e^N/A: not applicable.

^f^An expected result of random guess is 5.

^g^An expected result of random guess is 8.5.

### Determinants of Knowledge About DS

As depicted in Table 3, people less knowledgeable about DS were female, older, had a lower education status, lived in an area with fewer inhabitants, and earned less money per family member. Age appeared to be not linearly linked with knowledge about DS; people around 35 years were the most knowledgeable, and any increase or decrease in age from this point was linked to lower knowledge about DS in a nearly linear manner (such a curvilinear relationship was stable across the type of web-based service and after adjusting for potential confounders). Health-related habits were negligibly linked to knowledge about DS after adjusting for potential confounders. People who believed medicines were harmful or overprescribed by doctors had lower levels of knowledge about DS. Using a DS within the past 30 days was also an indicator of lower knowledge about DS. Among DS users, DS’s perceived beneficial effect was a negative modulator of knowledge about DS, whereas the harmful effect was a positive modulator. Trust in advertising DS appeared to be the strongest negative predictor of knowledge about DS, which was equal in both knowledge about DS domains. The effect of trust in advertising DS on knowledge about DS was particularly high among those who had contact with the respective advertisements. Interestingly, having contact with DS advertisements, without taking trust in advertising DS into account, had a somewhat positive effect on knowledge about DS. Being interested in DS was linked to a lower level of knowledge about DS. Although reported as the major source of knowledge about DS for web-based service users, internet and traditional media were negatively but weakly associated with knowledge about DS. Instead, the major source of false information regarding DS was found to be friends with no medical education. Among health care specialists, pharmacists appeared to be a misleading source of knowledge about DS.

High compatibility was found between nonparametric and parametric tests for the sensitivity analysis of the associations reported in Table 3 (the correlation of Spearman rho with Pearson *r* coefficients in univariate analyses was *r*=0.9985; 95% CI 0.9976-0.9990, and the median absolute difference between the corresponding coefficients was 0.004 of a maximum value of 0.034). Sensitivity analysis performed using GLM methods in a database of complete cases only (n=5633) was also highly consistent with the analysis performed in the original database following missing data imputation (the correlation of corresponding β regression coefficients was *r*=0.9991; 95% CI 0.9988-0.9993, and the median absolute difference between the corresponding coefficients was 0.003 of a maximum value of 0.018).

**Table 3:** Association between characteristics of the study participants and their knowledge about dietary supplements. Analyses were performed in the total sample of 6273 internet users. The results presented in italics are statistically significant at the Benjamini-Hochberg-corrected significance level of 0.036.

Characteristics		Raw analyses^a^							Adjusted analyses^b^					
		Knowledge about DS^c^–general		Knowledge about DS–specific		Knowledge about DS–total		Knowledge about DS–general			Knowledge about DS–specific		Knowledge about DS–total	
		Value, β coefficient (95% CI)	*P* value	Value, β coefficient (95% CI)	*P* value	Value, β coefficient (95% CI)	*P* value	Value, β coefficient (95% CI)		*P* value	Value, β coefficient (95% CI)	*P* value	Value, β coefficient (95% CI)	*P* value
**Sociodemographic**														
	Age^d^	−*.16 (−.18 to −.13)*	*<.001*	−*.26 (−.28 to −.24)*	*<.001*	−*.25 (−.28 to −.23)*	*<.001*	−*.05 (−.08 to −.03)*		*<.001*	−*.12 (−.15 to −.10)*	*<.001*	−*.11 (−.13 to −.08)*	*<.001*
	|Age−35|	−*.19 (−.22 to −.17)*	*<.001*	−*.23 (−.26 to −.21)*	*<.001*	*−.26 (−.28 to −.24)*	*<.001*	−*.10 (−.13 to −.08)*		*<.001*	−*.13 (−.16 to −.11)*	*<.001*	−*.14 (−.17 to −.12)*	*<.001*
	Sex (0=female and 1=male)	*.22 (.20 to .25)*	*<.001*	*.22 (.19 to .24)*	*<.001*	*.27 (.24 to .29)*	*<.001*	*.09 (.06 to .12)*		*<.001*	−.00 (−.03 to .03)	<.001	*.06 (.03 to .08)*	*<.001*
	Education	*.13 (.11 to .16)*	<.001	−.00 (−.03 to .02)	.89	*.08 (.06 to .11)*	*<.001*	*.11 (.08 to .13)*		<.001	−.02 (−.05 to .00)	.08	*.06 (.03 to .08)*	*<.001*
	Number of inhabitants	*.11 (.09 to .14)*	*<.001*	*.07 (.05 to .09)*	*<.001*	*.11 (.09 to .14)*	*<.001*	*.06 (.03 to .08)*		*<.001*	*.04 (.01 to .06)*	*.002*	*.06 (.04 to .08)*	*<.001*
	Earnings	*.19 (.16 to .21)*	*<.001*	*.14 (.12 to .16)*	*<.001*	*.20 (.18 to .23)*	*<.001*	*.05 (.02 to .07)*		*<.001*	.02 (−.01 to .05)	.13	*.04 (.02 to .07)*	*<.001*
**Health-related**														
	Health status	*.06 (.03 to .08)*	*<.001*	*.08 (.05 to .10)*	*<.001*	*.08 (.06 to .11)*	*<.001*	−*.03 (−.05 to −.00)*		*.02*	.01 (−.02 to .03)	.67	−.01 (−.04 to .01)	.22
	Diet	−*.06 (−.08 to −.03)*	*<.001*	−*.10 (−.12 to −.08)*	*<.001*	−*.09 (−.12 to −.07)*	*<.001*	.01 (−.01 to .04)		.36	.00 (−.02 to .02)	.96	.01 (−.02 to .03)	.53
	Physical activity	−*.03 (−.05 to −.00)*	*.03*	−*.03 (−.06 to −.01)*	*.01*	−*.04 (−.06 to −.01)*	*.05*	−*0.03 (−.05 to −.00)*		*.03*	−.01 (−.03 to .02)	.54	−.02 (−.04 to .00)	.07
	Current cigarette smoker	−.01 (−.03 to .02)	.44	.02 (−.01 to .04)	.13	.01 (−.02 to .03)	.68	−*.03 (−.05 to −.00)*		*.02*	−.02 (−.04 to .00)	.08	−*.03 (−.05 to −.01)*	*.01*
	Past but not current cigarette smoker	.04 (.02 to .07)	*<*.001	−.01 (−.04 to .01)	.33	.02 (−.00 to .04)	.11	*0.03 (.01 to .06)*		.006	−.03 (−.05 to −.00)	.03	.01 (−.02 to .03)	.61
	Current e-cigarette user	*.07 (.05 to .10)*	*<.001*	*.07 (.04 to .09)*	*<.001*	*.09 (.06 to .11)*	*<.001*	.02 (−.00 to .04)		.10	−.00 (−.03 to .02)	.74	.01 (−.01 to .03)	.37
	Past but not current e-cigarette user	*.04 (.02 to .07)*	*.001*	*.06 (.03 to .08)*	*<.001*	*.06 (.04 to .08)*	*<.001*	0.01 (−.01 to .03)		.45	0.01 (−.01 to .03)	.31	.01 (−.01 to .04)	.26
	Beliefs that medicines are overused	−*.08 (−.10 to −.05)*	*<.001*	−*.19 (−.21 to −.16)*	*<.001*	−*0.16 (−.19 to −.14)*	*<.001*	−.01 (−.03 to .02)		.54	−*.11 (−.13 to −.09)*	*<.001*	−*.07 (−.09 to −.05)*	*<.001*
	Beliefs that medicines are harmful	−*.19 (−.21 to −.16)*	*<.001*	−*.17 (−.20 to −.15)*	*<.001*	−*.22 (−.24 to −.20)*	*<.001*	−*.11 (−.13 to −.09)*		*<.001*	−*0.09 (−.11 to −007)*	*<.001*	−*.12 (−.15 to −.10)*	*<.001*
**DS-related**														
	Use of DS	−*.11 (−.13 to −.08)*	*<.001*	−*.29 (−.32 to −.27)*	*<.001*	−*.24 (−.26 to −.22)*	*<.001*	−*.03 (−.06 to −.01)*		*.005*	−*.21 (−.23 to −.18)*	*<.001*	−*.14 (−.17 to −.12)*	*<.001*
	Positive effect of DS	−*.12 (−.14 to −.09)*	*<.001*	−*.27 (−.29 to −.24)*	*<.001*	−*.23 (−.26 to −.21)*	*<.001*	−*.06 (−.09 to −.04)*		*<.001*	−*.20 (−.22 to −.17)*	*<.001*	−*.16 (−.18 to −.13)*	*<.001*
	Positive effect of DS with adjustment for DS use	−*.09 (−.12 to −.06)*	*<.001*	−*.14 (−.17 to −.11)*	*<.001*	−*.14 (−.17 to −.11)*	*<.001*	−*.06 (−.09 to −.04)*		*<.001*	−*.12 (−.15 to −.09)*	*<.001*	−*.11 (−.14 to −.08)*	*<.001*
	Negative effect of DS	.02 (−.01 to .04)	.22	.01 (−.01 to .04)	.36	.02 (−.01 to .04)	.18	*.03 (.01 to .05)*		*.01*	*.03 (.00 to .05)*	*.03*	*.03 (.01 to .06)*	*.003*
	Negative effect of DS with adjustment for DS use	.03 (.00 to .05)	.04	*.04 (.02 to .07)*	*<.001*	*.04 (.02 to .07)*	*<.001*	*.03 (.01 to .06)*		*.006*	*.04 (.02 to .07)*	*<.001*	*.05 (.02 to .07)*	*<.001*
	Having contact with DS advertisements	.00 (−.02 to .03)	.88	−*.04 (−.06 to −.01)*	*.004*	−.02 (−.05 to .00)	.11	*.04 (.02 to .07)*		*<.001*	.01 (−.01 to .04)	.26	*.03 (.01 to .06)*	*.003*
	Trust in advertising DS	−*.39 (−.41 to −.37)*	*<.001*	−*.39 (−.41 to −.37)*	*<.001*	−*.48 (−.50 to −.46)*	*<.001*	−*.31 (−.33 to −.28)*		*<.001*	−*.29 (−.31 to −.27)*	*<.001*	−*.37 (−.39 to −.34)*	*<.001*
	Having contact with DS advertisements×trust in advertising DS^e^	−*.14 (−.23 to −.05)*	*.004*	−.06 (−.16 to .03)	.21	−*.12 (−.21 to −.03)*	*.007*	−*.14 (−.23 to −.04)*		*.004*	−.06 (−.15 to .03)	.18	−*.12 (−.21 to −.04)*	*.005*
	Interest in DS	−*.11 (−.13 to −.08)*	*<.001*	−*.28 (−.30 to −.26)*	*<.001*	−*.24 (−.26 to −.21)*	*<.001*	−.01 (−.03 to .02)		.61	−*.17 (−.19 to −.14)*	*<.001*	−*.10 (−.13 to −.08)*	*<.001*
	Getting knowledge about DS from medical doctors^f^	−.01 (−.04 to .02)	.38	−.01 (−.04 to .01)	.31	−.02 (−.04 to .01)	.25	.00 (−.02 to .03)		.73	.01 (−.02 to .03)	.49	.01 (−.02 to .03)	.52
	Getting knowledge about DS from pharmacists^f^	−*.08 (−.11 to −.05)*	*<.001*	−*.09 (−.11 to −.06)*	*<.001*	−*.10 (−.13 to −.07)*	*<.001*	−.02 (−.05 to .01)		.18	−*.03 (−.05 to −.00)*	*.03*	−*.03 (−.05 to −.00)*	*.03*
	Getting knowledge about DS from dieticians^f^	.02 (−.01 to .04)	.23	−.01 (−.04 to .01)	.33	.00 (−.02 to −.03)	.84.	.00 (−.02 to .03)		.74	−.02 (−.04 to .01)	.21	−.01 (−.03 to .02)	.59
	Getting knowledge about DS from friends^f^	−*.09 (−.12 to −.07)*	*<.001*	−*.14 (−.16 to −.11)*	*<.001*	−*.14 (−.16 to −.11)*	*<.001*	−*.09 (−.11 to −.06)*		*<.001*	−*.13 (−.15 to −.11)*	*<.001*	−*.13 (−.15 to −.11)*	*<.001*
	Getting knowledge about DS from media^f^	−.02 (−.04 to .01)	.20	−*.16 (−.18 to −.13)*	*<.001*	−*.10 (−.13 to −.08)*	*<.001*	*.03 (.01 to .06)*		*.006*	−*.09 (−.12 to −.07)*	*<.001*	−*.03 (−.06 to −.01)*	*.003*

^a^Performed only with the variables reported.

^b^Adjusted for |Age−35|, sex, education, number of inhabitants, earnings, type of web-based service, and calendar year—all included as linear factors.

^c^DS: dietary supplement.

^d^Adjusted analyses do not include |Age−35|; the asociation between Age and Knowledge about dietary supplements seemed to be an inverted-U shape with a maximum in knowledge for age of about 35 years; consequently, Age was transformed in further analyses to |Age−35| to approximate the association to linear; the assoction between Age and Trust in advertising dietary supplements similarly looked as a nonlinear U shape with a minimum of trust for age of about 30 years.

^e^Adjusted for contact with dietary supplements advertisements and trust in advertising dietary supplements; having contact with dietary supplements advertisements in this model associated with knowledge about dietary supplements total in adjusted analysis with β of .11 (95% CI .04 to .18), *P=*.001; Moreover, trust in advertising DS associated with knowledge about dietary supplements—total in adjusted analysis among those who reported having contact with dietary supplements advertisements with β of −.38 (95% CI −.41 to −.36), *P<*.001, and those who did not report having contact with β of −.24 (95% CI −.31 to −.18), *P<*.001.

^f^The associations of knowledge about dietary supplements with getting knowledge about dietary supplements from a particular source were adjusted for all other sources of knowledge about dietary supplements.

### Prediction Model of Knowledge About DS

The variables selected for knowledge about DS modeling were trust in advertising DS, BMQ Harm, Age (following transformation to |Age−35|), Sex, and Getting knowledge about DS from friends. The model explained 31.2% of the variance in knowledge about DS. The predictors in the model were intercorrelated to a low extent, with a median absolute correlation coefficient of 0.114 and a maximum of 0.214 for trust in advertising DS and sex. The assumption of a linear contribution of predictors to the model was satisfactory. The model characteristics are listed in Table 4. According to this model, knowledge about DS can be predicted using the following formula:


Knowledge about DS = 19.397539 − 0.412775 × Trust in advertising DS − 0.23657 × BMQ Harm − 0.113879 × |Age − 35| + 1.850937 × Sex − 0.691575 × Getting knowledge about DS from friends

In the formula, *Trust in advertising DS* represents trust in advertising DS operationalized with the questionnaire on trust in advertising DS, ranging from 8 to 40; *BMQ Harm* represents BMQ operationalized with the BMQ questionnaire, subscale Harm, ranging from 4 to 20; *|Age−35|* is the absolute value of the difference between age (years) and 35; *Sex* was operationalized as 0 for females and 1 for males; and *Getting knowledge about DS* *from friends* represents a self-reported declaration on the extent of getting knowledge about DS from friends with no medical education (operationalized as 0 for *not at all*, 1 for *to little extent*, 2 for *to medium extent*, and 3 for *to large extent*).

**Table 4:** Characteristics of a model to describe knowledge about dietary supplements. The total sample of 6273 internet users was included. Test statistics, *P* value and coefficient of determination of a full model are *F*(5,6267)=568.2; *P*<.001; R^2^=0.312, respectively.

Predictor	Effect size				*F* test (*df*)		*P* value
	β coefficient	95% CI	pη^2^ (%)^a^				
Trust in advertising DS^b^	−.39	−.41 to −.37	16.4	123.4 (1,6267)		<.001	
Beliefs that medicines are harmful	−.13	−.15 to −.11	2.2	143.1 (1,6267)		<.001	
|Age−35|	−.17	−.19 to −.15	3.9	251.2 (1,6267)		<.001	
Sex (0=female and 1=male)	.15	.13 to .17	2.9	187.7 (1,6267)		<.001	
Getting knowledge about DS from friends	−.08	−.11 to −.06	1.0	62.0 (1,6267)		<.001	

^a^pη^2^: partial eta-squared.

^b^DS: dietary supplement.

**Figure 1:**
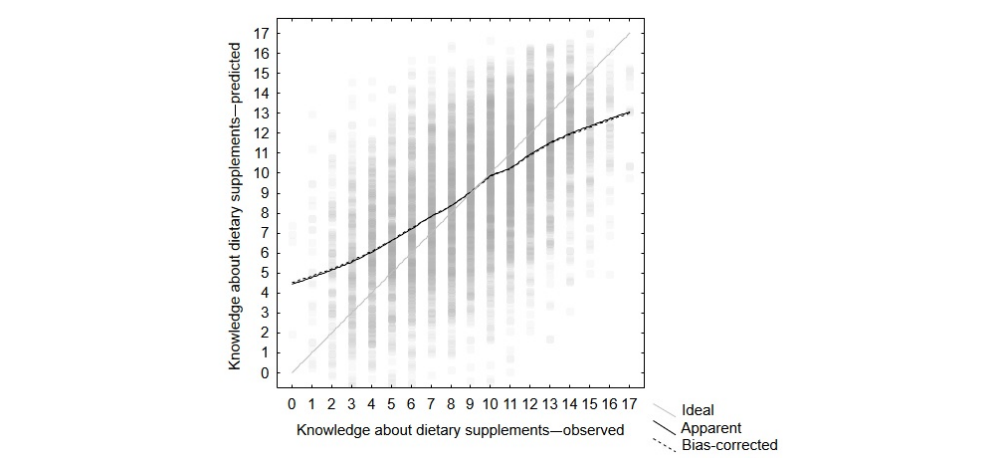
Calibration plot of the model to describe knowledge about dietary supplements. Black lines represent lowess (locally weighted scatterplot smoothing) fitted curves with a smoothing parameter of 0.25. The solid black line denotes the original (apparent) model, whereas the dotted black resulted from cross-validation (bias-corrected). The gray diagonal line represents hypothetical ideal prediction. Gray-filled circles depict individual data points predicted by the bias-corrected model. The circles are partially transparent; thus, the intensity of gray shadings reflects the density of points in an area.

The model could predict knowledge about DS with an MAE of 2.52 and an RMSE of 3.19 points (median 2.09, 1st to 3rd quartile 1.00-3.60). Although this performance was far from ideal, it was significantly better than a random guess based solely on knowledge about DS distribution that yielded MAE of 3.82 and RMSE of 4.76 points (median 3.23, 1st to 3rd quartile 1.59-5.49). Results of the model internal validation indicated that predictions of a bias-corrected model almost overlapped the original model with MAE of 2.51 and RMSE of 3.17 points (median 2.09, 1st to 3rd quartile 1.00-3.58), and the correlation between knowledge about DS values predicted by the original model and the bias-corrected one was very close (*r*=0.99986; 95% CI 0.99985-0.99986). Extreme values predicted by the bias-corrected model did not exceed an upper limit of 17 points; however, a lower limit was below zero due to the *Age* component not being restricted (36, ie, 0.57% of observations were below zero, with a minimum of −5.76). The model could predict relatively well the knowledge about DS values around its central value of 8.99 but exhibited worse performance with extreme knowledge about DS values, which was reflected by the deviation of a calibration plot from linearity. A calibration plot of the model is shown in Figure 1.

The 5 predictors could model not only knowledge about DS–total but also knowledge about DS–general and knowledge about DS–specific separately, with each of the predictors making a significant contribution to modeling both constructs, reaching determination coefficients of 20.4% and 22.0%, respectively. Importantly, although the model did not include as a predictor the type of web-based service through which a study participant accessed the survey, the model could predict (though overestimate) that the health service users had lower knowledge about DS than the users of the social networking service (7.6, 95% CI 7.5-7.7 vs 11.5, 95% CI 11.4-11.6, respectively; *P<*.001; a bias-corrected model). Moreover, the knowledge about DS score predicted by the bias-corrected model was found to be associated with the other participant characteristics examined in this study in a similar pattern as the associations of the observed knowledge about DS scores. The link between both associations, as expressed by Pearson *r*, was 0.95 (95% CI 0.91-0.97); the median absolute difference between the corresponding β coefficients was 0.03 (1st to 3rd quartile 0.01-0.09), and the maximum absolute difference was 0.38. Associations between knowledge about DS, predicted with the biased-corrected model and characteristics of the study participants, are reported in Multimedia Appendix 7.

### Knowledge About DS in Selected Subpopulations

Knowledge about DS was determined in some subpopulations thought to be critical to public health: older people aged 60 years or more, residents of rural areas (below 5000 inhabitants), and people with low earnings (below 1000 PLN [US $256] of monthly net household earnings per family member). All these subpopulations were found to have lower knowledge about DS than the remaining internet users. However, after adjusting for potential confounders, the effect persisted only for the older people and, to some extent, rural residents. For people with low earnings, the effect shrank to insignificant, as explained by confounders. The analysis of knowledge about DS in subpopulations is presented in Table 5. The proposed 5-predictor model to describe knowledge about DS worked well for rural residents and people with low earnings: all the predictors significantly contributed to prediction and presented a similar prediction error to other people. On the other hand, knowledge about DS among older people was not efficiently predicted by the 5-predictor model, with only trust in advertising DS and Age contributing significantly. The model performance according to subpopulations is presented in Table 5.

**Table 5:** Knowledge about dietary supplements and the model performance in subpopulations.

Type of analysis or model characteristics					Subpopulation				
					Older people (≥60 years; n=695)		Rural residents (<5000 inhabitants; n=783)		People with low earnings^a^ (<1000 PLN^b^ [US $256]; n=369)
**Difference in knowledge about DS**^**c**^–**total between a subpopulation and the remaining internet users**									
	**Raw difference^d^**								
		Values, mean		−2.1		−0.5		−1.0	
		95% CI		−2.4 to −1.8		−0.8 to −0.3		−1.3 to −0.6	
		*P* value		*<*.001		*<*.001		*<*.001	
	**Adjusted difference^e^**								
		Values, mean		−1.1		−0.3		−0.2	
		95% CI		−1.3 to −0.9		−0.5 to −0.0		−0.5 to 0.1	
		*P* value		*<*.001		.02		.18	
**Performance of the proposed 5-predictor model in a subpopulation**									
	**Predictors evaluation**								
		**Trust in advertising DS**							
			Values, β coefficient	−.34		−.44		−.37	
			95% CI	−.42 to −.27		−.50 to −.38		−.46 to −.28	
			*P* value	*<*.001		*<*.001		*<*.001	
		**Beliefs that medicines are harmful**							
			Values, β coefficient	−.06		−.18		−.15	
			95% CI	−.13 to .01		−.24 to −.12		−.24 to −.06	
			*P* value	.08		*<*.001		.002	
		**|Age−35|**							
			Values, β coefficient	−.10		−.13		−.20	
			95% CI	−.18 to −.03		−.19 to −.08		−.29 to −.11	
			*P* value	*<*.001		*<*.001		*<*.001	
		**Sex (0=female and 1=male)**							
			Values, β coefficient	.01		.11		.13	
			95% CI	−.06 to .08		.05 to .17		.04 to .22	
			*P* value	.76		.001		.005	
		**Getting knowledge about DS from friends**							
			Values, β coefficient	−.04		−.07		−.14	
			95% CI	−.11 to −.03		−.12 to −.01		−.23 to −.05	
			*P* value	.31		.03		.002	
	**Other indices**								
		**Full model statistics**							
			*F* test (*df*)	2.4 (5,689)		N/A^f^		N/A	
			*F* test (*df*)	N/A		8.2 (5,777)		N/A	
			*F* test (*df*)	N/A		N/A		28.1 (5,363)	
			*P* value	*<*.001		*<*.001		*<*.001	
		R-squared		0.129		0.340		0.279	
		MAE^g,h^		2.96		2.51		2.52	
		RMSE^g,i^		3.70		3.17		3.25	

^a^Monthly net household earnings per family member.

^b^PLN: Polish złoty. PLN was converted to US$ according to the average exchange rate on the study beginning date (source: Narodowy Bank Polski).

^c^DS: dietary supplements.

^d^Performed only with the variables reported.

^e^Adjusted for |Age-35|, sex, education, number of inhabitants, earnings, type of web-based service, calendar year—all expressed as linear factors—with the exclusion of a variable differentiating a subpopulation.

^f^N/A: not applicable.

^g^Assessed in a biased-corrected model.

^h^MAE: mean absolute error of prediction.

^i^RMSE: root mean squared error.

## Discussion

### Principal Findings

There is an ongoing debate over DS [49,50]. Recently, in Poland, several influential high-quality reports suggested unsatisfactory control over the DS market, resulting in inadequate safety [21,22,51]. These reports highlight the need for adequate public education to make informed decisions regarding DS intake [21,22]. This study fits into this discourse by examining knowledge about DS among Polish internet users. The results indicate the level of knowledge to be low and not much different from the previous preliminary report, which used the same research tool [38]. Several characteristics were found to be associated with knowledge about DS, with trust in advertising DS being the most influential; this study outlines a predictive model that could explain almost a third of the variance in knowledge about DS.

Although the results indicate the level of knowledge to be low, such a pessimistic diagnosis appears incomplete. A low level of knowledge about DS is not only derived from the unavailability of knowledge or ignorance; it should be interpreted rather as holding a *false belief* [52] that DS are thoroughly controlled, well tested, effective, and harmless or an expression of the overall confidence in DS properties, as outlined by the other authors [3,25,53]. A qualitative study aimed at exploring beliefs about DS and their use confirmed such interpretation as it identified that beliefs about DS people hold are largely related to their definition, effectiveness in health enhancement and illness prevention, and risks [54], which are the areas covered by the knowledge about DS questionnaire used in this study.

Our results indicate that the users of a web-based health service presented false beliefs about DS to a greater extent than the users of a web-based social networking service. This can be explained in at least three ways. First, health services may attract people who believe DS are effective and safe, as confidence in a product coexists with seeking information about it [55]. Second, web-based health services may not be efficient in promoting evidence-based facts regarding DS, which is obscured by advertising [56]. Third, social networking services users may be influenced to a strong degree by the nature of this environment [57] and may hence not fully engage with the survey and provide biased responses.

In general, people with low knowledge about DS presented such characteristics as interest in the topic of DS, tendency to seek information about them and eagerness to know more, use of DS, and subjective experience of their positive effects without negative effects. The literature on the relationship between DS intake and knowledge about DS appears confusing, with some of the studies reporting positive links [30] and others negative links [38]. However, this is only an apparent discrepancy, as the result depends on the method used. DS users seem to present high *subjective* awareness and confidence in DS [25], which are, in fact, *objectively* false beliefs [33]. The major reason why subjective and objective knowledge are not in line was outlined by the Dunning-Kruger effect [58], stating that unknowledgeable people tend to overestimate their ability [53]. However, the reason why some people do not achieve high scores in objective knowledge about DS, despite the efforts made, should be sought in the quality of information sources they use to learn about DS. Popular beliefs about DS are in conflict with evidence-based facts. Media coverage, particularly DS advertisements, often reinforces popular beliefs rather than providing facts [10-15]. For example, Wierzejska [59], while analyzing the Polish DS market, found that most DS advertisements promise beneficial effects to the human body, going beyond the standards established for food. Trusting such messages may contribute to the accumulation of false beliefs regarding DS.

trust in advertising DS was found to be the strongest among the examined predictors of knowledge about DS and was expressed in a negative way. trust in advertising DS was particularly negatively linked with knowledge about DS among those who reported having contact with DS advertisements recently; however, it could associate with knowledge about DS even among those who did not. Interestingly, having contact with DS advertisements itself, irrespective of trust in advertising DS, was weakly associated with knowledge about DS, but in a positive way. Developing trust, particularly in its cognitive form, is a long-lasting process that requires deeper commitment than just *having a contact* [60,61]. The DS consumer who trusts advertisements is *dependent* on a message and therefore exposed to a significant risk [62] of being misinformed by DS advertisements, which spread unverified and misleading claims regarding health benefits of DS [59]. On the other hand, having contact with DS advertisements appears incapable of deceiving consumers. Moreover, being chronically exposed to DS advertisements may result in the opposite effect of advertising fatigue [63], leading to advertising distrust, which may, in turn, promote prudence and true beliefs regarding DS.

Causal relationship between knowledge about DS and trust in advertising DS is not evident, although possible [38]. The idea of gaining false beliefs about DS in response to high trust in advertising DS is particularly attractive to opponents of advertising. This is to some extent supported by our findings, as people reporting to get information about DS from media presented lower knowledge about DS. On the other hand, the idea of trust in advertising DS being shaped as a result of holding false beliefs about DS appears similarly likely and may reflect the natural phenomenon of gaining confidence in messages that appear to be true [55].

This study found the media (magazines, television, radio, and the internet) to be the primary source of information about DS to internet users. This is not surprising regarding the nature of the population studied and is in line with some [4,31], but not all [28], previous studies. The top rank of media may be regarded as undermining the unwavering position of health care specialists in providing health-related information [3,64,65]. Considering that web-based resources about DS leave much to be desired [10-15] and are full of deceitful DS advertisements, one could suspect that using the internet as a source of information about DS blurs the knowledge about DS to a large degree. Strikingly, the link presents merely a small effect size, and for knowledge about DS–general, the association was even positive. Although some popular internet resources about DS suggest their therapeutic benefits (possibly contributing to lower knowledge about DS–specific), they present the true characteristics of the products, quoting the definition or introducing the regulatory framework (possibly contributing to an increase in knowledge about DS–general) [66-68]. Similarly, the association of *interest in DS* and *use of DS* was found to be negative only with knowledge about DS–specific but not (or almost not) with knowledge about DS–general. Finally, accessing web-based DS information should not be perceived as a source of criticism. Quite the opposite, it offers new opportunities to improve the patient-physician relationship and, in turn, verify and reinforce true beliefs about DS [69].

Concerns should be raised about the negative link between knowledge about DS and getting information about DS from friends with no medical education. Word-of-mouth advertising was suggested to be efficient in spreading ideas long ago [70]. It is considered more credible than a commercial and may be successfully applied to informal promotions of health-related products [71]. As internet users report experiencing positive, much more frequently than negative, DS effects (according to our results) and word-of-mouth is likely to transmit subjective—yet unproven—information, it may strengthen false beliefs about DS.

Among health care practitioners, only obtaining information about DS from pharmacists was associated with lower knowledge about DS. Although the effect size was very small, it may mean that pharmacist advice contributed to strengthening false beliefs about DS. This is particularly worrying, as pharmacists were reported as the second most important source of information about DS and the first personal one. Concerns related to the ethics of DS being sold in pharmacies have already been raised, and a conflict between dispensing and counseling on DS has been identified [72]. Yet this is the role of pharmacists to educate patients about these products [73].

Our results indicate a negative association between knowledge about DS and both subscales of the BMQ-General, implying a link between confidence in DS and overall negative views about medicines. This association reflects an existing conflict between alternative and conventional medicine [74,75]. This confirms previous findings that individuals who advocate alternative medicine distrust or are dissatisfied with conventional care [76-78]. Our results support the need for reconciliation and integration of both attitudes for the sake of patients [74,75].

Some sociodemographic determinants of knowledge about DS were identified in this study. Women exhibited lower knowledge about DS than men. However, the result was not replicated by other knowledge about DS studies, which found insignificant [23,25,38] or inconsistent [28] differences between sexes. The effect size of the difference in this study, although very small following adjustment for confounders, was significant. It could partially reflect the overall tendency for women to use DS more than men [1,6,28,79] or hold more positive attitudes toward advertising [80], associated with more confidence in DS and lower objective knowledge about DS, as discussed above. A detailed consideration of the reasons why women are apparently more willing to use DS is presented elsewhere [5]. This study also found that people who are generally better educated, living in larger places of residence, or having more money for living presented higher knowledge about DS, particularly in its general domain. A previous preliminary study using the same knowledge about DS measuring tool also reported such findings for the overall education status [38]. Better educated people tend to rely less on advertising [81,82], which is a factor linked to better knowledge about DS. This finding may support our results.

Age presented the most complicated pattern of a relationship with knowledge about DS, with its peak observed in people around 35 years. There is a scarcity of literature linking age to knowledge about DS. In the only study that could be mentioned, knowledge about DS was tested in the general population of young adults with a mean age of 25 years and an SD of 5 years. A subgroup of participants aged >31 years was able to provide the definition of supplementation (objective knowledge about DS) in a greater percentage in comparison with younger participants. Moreover, there was a trend for younger participants to express higher levels of subjective, possibly false knowledge [33]. Although the cited study is in line with this study concerning young adults, it did not include older people. Studies examining DS intake across various age groups suggested that older people used more DS [31,83], possibly having more false-positive beliefs about DS, or no significant difference was found [26,30]. It is not surprising that there is no clear replication of these results in the literature, as the knowledge about DS–age pattern revealed is not linear and would require an explorative research approach and a large sample size. Obtaining such results was not possible until this study was performed. Research on advertising attitudes may help to explain this phenomenon. Consumers aged 40 years or more were found to have a more positive attitude toward advertisements [81]. Older adults were also more likely to be persuaded by advertising messages [84]. In another study, a more favorable attitude was expressed by younger people aged less than 30 years [85]. None of these studies found a curvilinear relationship between age and susceptibility to advertising; however, their synthesis may approximate such an association. Interestingly, in this study, trust in advertising DS, which is a similar construct to the one considered above, also achieved its minimum in young adults around 30 years old and increased in both younger and older people. As trust in advertising DS is closely associated with knowledge about DS, it may contribute to the explanation of this phenomenon. On the other hand, not all the reports replicate the above findings [80], and such interpretation should not be regarded as straightforward.

In the culminating part of this study, a predictive model of knowledge about DS was built using a multivariate linear regression strategy. The model was able to explain almost one-third of the knowledge about DS variability and included 5 predictors. Although the choice of the predictors was based to some extent on statistical significance, the most objective characteristics outlined a priori in the study protocol [39] were reflected in the final model, which makes it partially based on subject matter knowledge as generally advised [86]. The proposed model was simple enough not to be prone to overfitting, as tested by the internal validation procedure, and versatile enough to predict differences between the 2 web-based services tested and forecast most of the associations tested. The model worked well in subpopulations of rural citizens and people of low income, but worse in older people, whose beliefs related to DS require special attention [26]. This is likely the model will predict the outcome not much worse in an external validation study and in practice. It also has the potential for electronic application. Although the model still made significant mistakes, leaving more than two-thirds of knowledge about DS variance unallocated, it may help scientists recognize further paths of research: health care workers identify people endangered with illusory beliefs about DS, and educators tailor campaigns to the public to induce behavioral change [20,87,88].

The issue of generalizability requires some debate before any conclusions are drawn. First, a convenience sample of internet users from a restricted number of services may present a barrier to these findings’ generalizability to the entire web-based community. Health and social networking services presented extreme demographics, likely covering the scope of internet users in general. Considering the relatively large number of possibly not overlapping users of both services [47], this study could capture a broad range of internet users in Poland. Labeling the sample as representative, however, should be avoided. Similarly, the sample should not be regarded as reflecting the overall Polish population. Although most young people access the internet, more than three-fourths of Poles aged 65 years or more remain offline [89], and the group may represent different attitudes toward DS [26].

Apart from generalizability issues, this study has some limitations that warrant mention while interpreting the results. First, the results come from self-reported declarative data, which may be shifted according to the social desirability bias theory [90]. Such results may only approximate the true beliefs and behaviors of study participants and require cautious interpretation [91]. Second, the study did not include external validation of the findings, particularly the predictive model of knowledge about DS. External validation is essential before implementing the model in practice [92]. Third, although single-item measures have the potential to accurately reflect the construct assessed [93], some of the measures in this study were not rigorously validated, although they were used in the same form elsewhere [25]. Fourth, the study did not examine other potential knowledge about DS predictors such as general medical knowledge [94], experience with particular DS products, or personality traits. The survey did not include these variables to keep it short and to minimize the burden of responding [95]. This study, however, substantially reduced possible bias through prospective registration of the study protocol, using some valid research instruments, reporting data transparently, and performing sensitivity analyses.

This report requires further testing to establish the results’ external validity among different groups of internet users to account for their diversity. Further investigation into the mechanisms explaining the relationships between the analyzed constructs is needed; this should be supplemented with more qualitative research to confirm the obtained results and reveal a deeper understanding of the problem. Finally, this and any follow-up studies should aim to develop and implement an effective strategy for public education regarding DS, ideally in a web-based form, to ensure rational use of the products for the public’s greatest benefit.

### Conclusions

Internet users in Poland with no medical education, particularly those attending a health service, exhibit some false beliefs regarding the quality requirements, efficacy, and safety of DS. Holding such false beliefs is positively associated with trusting DS advertising; their intake, particularly when positive effects are subjectively experienced; being interested in the issues of DS; getting information about them through word-of-mouth; beliefs that medicines are harmful or overused; and some demographic characteristics. The proposed 5-predictor model to forecast knowledge about DS can explain almost a third of its variability and appears resistant to overfitting. The results call for implementing efficient education about DS, including the promotion of advertising literacy, to correct consumers’ attitudes toward DS. The results also underline the need to introduce more conservative regulatory frameworks for DS marketing. Further research is needed for external validation of the results and to obtain a more comprehensive understanding of the revealed phenomena.

## Appendix

Multimedia Appendix 1 Modifications in the study protocol made after the study commencement.

Multimedia Appendix 2 Statements in the questionnaire on knowledge about dietary supplements and analysis of the items.

Multimedia Appendix 3 The survey.

Multimedia Appendix 4 Database cleaning procedure and results.

Multimedia Appendix 5 Data missingness analysis.

Multimedia Appendix 6 Data missingness analysis—continued.

Multimedia Appendix 7 Associations of knowledge about dietary supplements predicted with the biased-corrected model with characteristics of the study participants.

## Abbreviations

BMQ: beliefs about medicines questionnaire
DS: dietary supplement
GLM: general linear model
MAE: mean absolute error
RMSE: root mean squared error

## References

[1] Kantor ED, Rehm CD, Du M, White E, Giovannucci EL (2016). Trends in dietary supplement use among US adults from 1999-2012. J Am Med Assoc.

[2] Kamangar F, Emadi A (2012). Vitamin and mineral supplements: do we really need them?. Int J Prev Med.

[3] Sirico F, Miressi S, Castaldo C, Spera R, Montagnani S, Meglio FD, Nurzynska D (2018). Habits and beliefs related to food supplements: results of a survey among Italian students of different education fields and levels. PLoS One.

[4] Bailey RL, Gahche JJ, Miller PE, Thomas PR, Dwyer JT (2013). Why US adults use dietary supplements. JAMA Intern Med.

[5] Conner M, Kirk SF, Cade JE, Barrett JH (2001). Why do women use dietary supplements? The use of the theory of planned behaviour to explore beliefs about their use. Soc Sci Med.

[6] Dickinson A, Blatman J, El-Dash N, Franco JC (2014). Consumer usage and reasons for using dietary supplements: report of a series of surveys. J Am Coll Nutr.

[7] Loiacono C, Palermi S, Massa B, Belviso I, Romano V, Gregorio AD, Sirico F, Sacco AM (2019). Tendinopathy: pathophysiology, therapeutic options, and role of nutraceutics. A narrative literature review. Medicina (Kaunas).

[8] Meglio FD, Sacco A, Belviso I, Romano V, Camargo F, Loiacono C, Palermi S, Pempinello C, Montagnani S, Nurzynska D, Castaldo C (2020). Influence of supplements and drugs used for the treatment of musculoskeletal disorders on adult human tendon-derived stem cells. Muscle Ligaments and Tendons J.

[9] Gabriels G, Irhuma M (2019). The potential impact of dietary supplement adulteration on patient assessment and treatment from a healthcare provider’s perspective. S Afr Fam Pract.

[10] Baudischova L, Straznicka J, Pokladnikova J, Jahodar L (2018). The quality of information on the internet relating to top-selling dietary supplements in the Czech Republic. Int J Clin Pharm.

[11] Jordan MA, Haywood T (2007). Evaluation of internet websites marketing herbal weight-loss supplements to consumers. J Altern Complement Med.

[12] Palmour N, Vanderbyl BL, Zimmerman E, Gauthier S, Racine E (2013). Alzheimer's disease dietary supplements in websites. HEC Forum.

[13] Zhao Y, Zhang J (2017). Consumer health information seeking in social media: a literature review. Health Info Libr J.

[14] Schenker Y, Arnold RM, London AJ (2014). The ethics of advertising for health care services. Am J Bioeth.

[15] Benigeri M, Pluye P (2003). Shortcomings of health information on the internet. Health Promot Int.

[16] Cohen PA (2016). The supplement paradox: negligible benefits, robust consumption. J Am Med Assoc.

[17] Myung SK, Kim Y, Ju W, Choi HJ, Bae WK (2010). Effects of antioxidant supplements on cancer prevention: meta-analysis of randomized controlled trials. Ann Oncol.

[18] White CM (2020). Dietary supplements pose real dangers to patients. Ann Pharmacother.

[19] Sligo FX, Jameson AM (2000). The knowledge?behavior gap in use of health information. J Am Soc Inf Sci.

[20] de Vries Hein, Pajor E, Curfs K, Eggers S, Oenema A (2019). How to foster informed decision making about food supplements: results from an international Delphi study. Health Educ Res.

[21] (2019). Poland and supplements: opportunities for European leadership. Polish Economic Institute.

[22] (2017). NIK o dopuszczaniu do obrotu suplementów diety - Najwyższa Izba Kontroli. Supreme Audit Office.

[23] Aina BA, Ojedokun OA (2014). Knowledge and use of dietary supplements by students of College of Medicine, University of Lagos, Idi-Araba, Lagos, Nigeria. J Basic Clin Pharm.

[24] Axon DR, Vanova J, Edel C, Slack M (2017). Dietary supplement use, knowledge, and perceptions among student pharmacists. Am J Pharm Educ.

[25] Carvey CE, Farina EK, Lieberman HR (2012). Confidence in the efficacy and safety of dietary supplements among United States active duty army personnel. BMC Complement Altern Med.

[26] Marinac JS, Buchinger CL, Godfrey LA, Wooten JM, Sun C, Willsie SK (2007). Herbal products and dietary supplements: a survey of use, attitudes, and knowledge among older adults. J Am Osteopath Assoc.

[27] Molinero O, Márquez S (2009). Use of nutritional supplements in sports: risks, knowledge, and behavioural-related factors. Nutr Hosp.

[28] Sharma A (2014). Knowledge, attitude and practices related to dietary supplements and micronutrients in health sciences students. J Clin Diagn Res.

[29] Žeželj S, Tomljanović A, Jovanović G, Krešić G, Peloza O, Dragaš-Zubalj N, Prokurica I (2018). Prevalence, knowledge and attitudes concerning dietary supplements among a student population in Croatia. Int J Environ Res Public Health.

[30] Algaeed HA, AlJaber MI, Alwehaibi AI, AlJaber LI, Arafah AM, Aloyayri MA, Binsebayel OA, Alotaiq SA, Alfozan MA, Ahmed IB (2019). General public knowledge and use of dietary supplements in Riyadh, Saudi Arabia. J Family Med Prim Care.

[31] Alowais MA, Selim MAEH (2019). Knowledge, attitude, and practices regarding dietary supplements in Saudi Arabia. J Family Med Prim Care.

[32] Dodge T, Litt D, Kaufman A (2011). Influence of the dietary supplement health and education act on consumer beliefs about the safety and effectiveness of dietary supplements. J Health Commun.

[33] Kołodziej G, Cyran-Grzebyk B, Majewska J, Kołodziej K (2019). Knowledge concerning dietary supplements among general public. Biomed Res Int.

[34] Clement J Global digital population as of July 2020. Statista.

[35] Kwan D, Hirschkorn K, Boon H (2006). U.S. and Canadian pharmacists' attitudes, knowledge, and professional practice behaviors toward dietary supplements: a systematic review. BMC Complement Altern Med.

[36] Parmenter K, Wardle J (1999). Development of a general nutrition knowledge questionnaire for adults. Eur J Clin Nutr.

[37] Steyn NP, Labadarios D, Nel JH, Heidi-Lee R (2005). Development and validation of a questionnaire to test knowledge and practices of dietitians regarding dietary supplements. Nutrition.

[38] Karbownik MS, Paul E, Nowicka M, Nowicka Z, Kowalczyk RP, Kowalczyk E, Pietras T (2019). Knowledge about dietary supplements and trust in advertising them: development and validation of the questionnaires and preliminary results of the association between the constructs. PLoS One.

[39] Karbownik MS Determinants of knowledge about and use of dietary supplements. OSFHome.

[40] von Elm E, Altman DG, Egger M, Pocock SJ, Gøtzsche PC, Vandenbroucke JP, STROBE Initiative (2008). The Strengthening the Reporting of Observational Studies in Epidemiology (STROBE) statement: guidelines for reporting observational studies. J Clin Epidemiol.

[41] Moons KGM, Altman DG, Reitsma JB, Ioannidis JPA, Macaskill P, Steyerberg EW, Vickers AJ, Ransohoff DF, Collins GS (2015). Transparent Reporting of a multivariable prediction model for Individual Prognosis or Diagnosis (TRIPOD): explanation and elaboration. Ann Intern Med.

[42] Hilton CE (2015). The importance of pretesting questionnaires: a field research example of cognitive pretesting the Exercise referral Quality of Life Scale (ER-QLS). Int J Soc Res Methodol.

[43] Karbownik MS, Jankowska-Polańska B, Horne R, Górski KM, Kowalczyk E, Szemraj J (2020). Adaptation and validation of the Polish version of the beliefs about medicines questionnaire among cardiovascular patients and medical students. PLoS One.

[44] Horne R, Weinman J, Hankins M (1999). The beliefs about medicines questionnaire: the development and evaluation of a new method for assessing the cognitive representation of medication. Psychol Health.

[45] Paxton AE, Strycker LA, Toobert DJ, Ammerman AS, Glasgow RE (2011). Starting the conversation performance of a brief dietary assessment and intervention tool for health professionals. Am J Prev Med.

[46] Biernat E, Stupnicki R, Gajewski A (2007). International Physical Activity Questionnaire (IPAQ) - Polish version. Physical Education and Sport.

[47] Badanie Gemius/PBI. SitePoint.

[48] Leiner DJ (2019). Too fast, too straight, too weird: post hoc identification of meaningless data in internet surveys. SSRN Journal.

[49] Dwyer JT, Coates PM, Smith MJ (2018). Dietary supplements: regulatory challenges and research resources. Nutrients.

[50] Starr RR (2015). Too little, too late: ineffective regulation of dietary supplements in the United States. Am J Public Health.

[51] Makowska M, Jasiński Ł (2019). A discussion of the unresolved 2016/17 plans for regulating the Polish dietary supplements market. Health Policy.

[52] Parikh R, Renero A, Floyd J, Bokulich A (2017). Justified true belief: plato, gettier, turing. Philosophical Explorations of the Legacy of Alan Turing; Boston Studies in the Philosophy and History of Science.

[53] Homer PM, Mukherjee S (2019). Lay theories and consumer perceptions of dietary supplements. J Consumer Behav.

[54] Pajor EM, Oenema A, Eggers SM, de Vries H (2017). Exploring beliefs about dietary supplement use: focus group discussions with Dutch adults. Public Health Nutr.

[55] Rong L, Kim J, Park J (2007). The effects of internet shoppers' trust on their purchasing intention in China. J Inf Technol Manag.

[56] Sun Y, Zhang Y, Gwizdka J, Trace CB (2019). Consumer evaluation of the quality of online health information: systematic literature review of relevant criteria and indicators. J Med Internet Res.

[57] Waheed H, Anjum M, Rehman M, Khawaja A (2017). Investigation of user behavior on social networking sites. PLoS One.

[58] Kruger J, Dunning D (1999). Unskilled and unaware of it: how difficulties in recognizing one's own incompetence lead to inflated self-assessments. J Pers Soc Psychol.

[59] Wierzejska R (2016). Whether the advertisement of dietary supplements is objective source of data about their impact on health? Analysis of broadcasting advertisements in the terms of the food law. Wiad Lek.

[60] Lane C, Bachmann R (1998). Trust within and between organizations.

[61] Zhang X, Zhang Q (2005). Online trust forming mechanism: approaches and an integrated model. Proceedings of the 7th international conference on Electronic commerce.

[62] Nooteboom B, Six F (2003). The trust process in organizations : empirical studies of the determinants and the process of trust development.

[63] Rao A, Miller P (1975). Advertising/sales response functions. J Advert Res.

[64] Hesse BW, Nelson DE, Kreps GL, Croyle RT, Arora NK, Rimer BK, Viswanath K (2005). Trust and sources of health information: the impact of the Internet and its implications for health care providers: findings from the first Health Information National Trends Survey. Arch Intern Med.

[65] Alduraywish SA, Altamimi LA, Aldhuwayhi RA, AlZamil LR, Alzeghayer LY, Alsaleh FS, Aldakheel FM, Tharkar S (2020). Sources of health information and their impacts on medical knowledge perception among the Saudi Arabian population: cross-sectional study. J Med Internet Res.

[66] PoradnikZdrowie PoradnikZdrowie.pl znów na czele - ABCzdrowie.pl, Medonet.pl i MP.pl w górę (TOP10 serwisów o zdrowiu). pl, Medonet.

[67] Suplementy diety - charakterystyka, składniki, odchudzanie. WP abcZdrowie.

[68] Do czego potrzebne są suplementy?.

[69] Tan SS-L, Goonawardene N (2017). Internet health information seeking and the patient-physician relationship: a systematic review. J Med Internet Res.

[70] Lazarfeld P, Merton R (1949). Mass communication, popular taste, and organized social action. Mass Communications.

[71] Holdford D (2005). Understanding the dynamics of the pharmaceutical market using a social marketing framework. J Consum Mark.

[72] Boon H, Hirschkorn K, Griener G, Cali M (2009). The ethics of dietary supplements and natural health products in pharmacy practice: a systematic documentary analysis. Int J Pharm Pract.

[73] Douglas PL, McCarthy H, McCotter LE, Gallen S, McClean S, Gallagher AM, Ray S (2019). Nutrition education and community pharmacy: a first exploration of current attitudes and practices in Northern Ireland. Pharmacy (Basel).

[74] Baars EW, Hamre HJ (2017). Whole medical systems versus the system of conventional biomedicine: a critical, narrative review of similarities, differences, and factors that promote the integration process. Evid Based Complement Alternat Med.

[75] Truant TL, Balneaves LG, Fitch MI (2015). Integrating complementary and alternative medicine into cancer care: Canadian oncology nurses' perspectives. Asia Pac J Oncol Nurs.

[76] Nahin RL, Dahlhamer JM, Stussman BJ (2010). Health need and the use of alternative medicine among adults who do not use conventional medicine. BMC Health Serv Res.

[77] Chao MT, Wade C, Kronenberg F (2008). Disclosure of complementary and alternative medicine to conventional medical providers: variation by race/ethnicity and type of CAM. J Natl Med Assoc.

[78] Egan B, Hodgkins C, Shepherd R, Timotijevic L, Raats M (2011). An overview of consumer attitudes and beliefs about plant food supplements. Food Funct.

[79] Foote JA, Murphy SP, Wilkens LR, Hankin JH, Henderson BE, Kolonel LN (2003). Factors associated with dietary supplement use among healthy adults of five ethnicities: the Multiethnic Cohort Study. Am J Epidemiol.

[80] Ahlluwalia S, Singh S (2020). Consumers’ demographics as predictors of their perception of online advertising: are they still relevant in the e-landscape?. Global Business Review.

[81] Milakovic IK, Mihić M (2015). Predictors and outcome of attitudes towards advertising: demographics, personal factors and wom.

[82] Shavitt S, Lowrey P, Haefner J (1998). Public attitudes toward advertising: more favorable than you might think. J Advert Res.

[83] Gahche JJ, Bailey RL, Potischman N, Dwyer JT (2017). Dietary supplement use was very high among older adults in the United States in 2011-2014. J Nutr.

[84] Phillips DM, Stanton JL (2004). Age-related differences in advertising: recall and persuasion. J Target Meas Anal Mark.

[85] Diehl S, Mueller B, Terlutter R, Rosengren S, Dahlén M, Okazaki S (2013). The influence of demographic factors on the perception of humane-oriented (CSR) appeals in advertisements: a multi-country analysis. Advances in Advertising Research (Vol. IV), The Changing Roles of Advertising.

[86] Wynants L, Collins GS, Van Calster B (2017). Key steps and common pitfalls in developing and validating risk models. Brit J Obstet Gynec.

[87] Chiba T, Kobayashi E, Okura T, Sekimoto M, Mizuno H, Saito M, Umegaki K (2020). An educational intervention improved knowledge of dietary supplements in college students. BMC Public Health.

[88] Begdache L, Kianmehr H, Heaney CV (2020). College education on dietary supplements may promote responsible use in young adults. J Diet Suppl.

[89] (2017). Internet use. Public Opinion Research Center.

[90] Latkin CA, Edwards C, Davey-Rothwell MA, Tobin KE (2017). The relationship between social desirability bias and self-reports of health, substance use, and social network factors among urban substance users in Baltimore, Maryland. Addict Behav.

[91] Steene-Johannessen J, Anderssen SA, van der Ploeg Hidde P, Hendriksen IJM, Donnelly AE, Brage S, Ekelund U (2016). Are self-report measures able to define individuals as physically active or inactive?. Med Sci Sports Exerc.

[92] Bleeker SE, Moll HA, Steyerberg EW, Donders ART, Derksen-Lubsen G, Grobbee DE, Moons KGM (2003). External validation is necessary in prediction research: a clinical example. J Clin Epidemiol.

[93] Loftfield E, Yi S, Immerwahr S, Eisenhower D (2015). Construct validity of a single-item, self-rated question of diet quality. J Nutr Educ Behav.

[94] Bachmann LM, Gutzwiller FS, Puhan MA, Steurer J, Steurer-Stey C, Gigerenzer G (2007). Do citizens have minimum medical knowledge? A survey. BMC Med.

[95] Egleston BL, Miller SM, Meropol NJ (2011). The impact of misclassification due to survey response fatigue on estimation and identifiability of treatment effects. Stat Med.

