# Supplementary material for: Determinants of Knowledge About Dietary Supplements Among Polish Internet Users: Nationwide Cross-sectional Study
Source: J Med Internet Res. 2021 Apr 21;23(4):e25228. doi: 10.2196/25228 (PMC8100877; doi:10.2196/25228)
Supplement: Multimedia Appendix 1 [file jmir_v23i4e25228_app1.pdf]

Supplementary File 1

Modifications in the study protocol made after the study commencement

The following modifications in the study protocol after the study commencement (26 November 2019) were done:

Record in the original protocol	Modification	Reason for modification
**Modifications of major importance**		
Cohort characteristics: “People potentially interested in health issues.”	Cohort characteristics: Internet users	1) Willingness to broaden the scope of the study and to compare the Internet users from different services. 2) Difficulty in getting the planed number of participants (10,000).
Source of sample: DOZ.pl media	Source of sample: DOZ.pl media, Wykop.pl (a decision to include this online service was made and implemented in the 16th day of the study)	
**Modifications of minor importance**		
Data analysis plan: “Variables with more than 50% of missing values and cases with more than 50% of missing values will be deleted from the database.”	Additionally to this, potentially meaningless records were identified according to the survey completion time (less than 2:30) and further deleted.	The researchers were unaware of availability of data on survey completion time (which is regarded one of the best indicators of potentially meaningless survey records) before the study start.
N/A	Variable transformation was considered before including a potential predictor to the KaDS model.	To allow the model better reflect the nature of a phenomenon.
Data analysis plan: “The associations will be reported as both unadjusted and adjusted for all the tested sociodemographic measures.”	Additionally to adjustment for sociodemographic measures, 1) a type of online service through which a study participant accessed the survey and 2) a calendar year in which a participant completed the survey were included as potential confounders.	1) To control the result of adjusted analyses over the variability resulted from the source of Internet user, which could not be planned originally. 2) To adjust for potential effect of “Broadcasting agreement about the rules and regulations for advertising dietary supplements” (self-regulation), which was signed in Poland and became effective on January 01, 2020.

Data analysis plan: “If the data is imputed (see point "3. g"), sensitivity analysis of univariate (unadjusted) associations between DVs and the remaining measures will be performed in the dataset of complete cases only to test the accuracy of missing data imputation”	Additionally to sensitivity analysis with unadjusted analyses, adjusted analyses were also included in the sensitivity analysis. Moreover, a sensitivity analysis was performed also with non-parametric tests, if applicable.	To examine potential bias resulted from occurrence of missing data and its imputation as well as statistical test selection.
**Modifications of insignificant importance**		
Inclusion criteria: “18 years of age or more”<br>Exclusion criteria: “Self-declared medical education”	People 18 years of age or more and with declared medical education were allowed to participate in the study, but were not in scope of its interest and consequently were removed from the database	Original intention was to let all the people (who communicate in Polish and express informed consent) participate in the study with subsequent exclusion of those not being in scope of interest. This was incorrectly expressed in the original study protocol.
Data analysis plan:<br>“The pattern of data missingness will be assessed with Little's test for data missing completely at random (MCAR). In case of significant violation of MCAR assumption, for each variable, the association of missingness with all the other variables will be examined.”	Little’s test was not performed and the data analysis were proceeded to test the extent of associations of missingness with all the other variables, irrespective of MCAR assumption violation.	The Little’s test was found unavailable in the latest version of R Software (4.0.0) and was assessed as redundant in the analysis.

N/A – not applicable

KaDS – knowledge about dietary supplements
