# Supplementary material for: Determinants of Knowledge About Dietary Supplements Among Polish Internet Users: Nationwide Cross-sectional Study
Source: J Med Internet Res. 2021 Apr 21;23(4):e25228. doi: 10.2196/25228 (PMC8100877; doi:10.2196/25228)
Supplement: Multimedia Appendix 2 [file jmir_v23i4e25228_app2.pdf]

Supplementary File 2

Statements in the Questionnaire on Knowledge about Dietary Supplements and analysis of the items

Table. Numbers and frequencies of correct answers given to the items of the Questionnaire on Knowledge about Dietary Supplements

Item number	Statement	True (T) or False (F)	General (G) or Specific (S) subscale	Correct answers	
				Number	Frequency [%]
1	Taking vitamin and mineral supplements prevents diseases in healthy people.	F	S	3674	58.57
2	The quality of dietary supplements is routinely tested before being marketed.	F	G	3867	61.65
3	People with kidney disease should not use high doses of vitamin C.	T	S	4542	72.41
4	The use of multivitamin preparations protects against heart diseases.	F	S	5125	81.70
5	An ingredient may be sold both as a medicine or as a dietary supplement.	T	G	4235	67.51
6	Taking excessive amounts of magnesium supplements can cause diarrhea and nausea.	T	S	5146	82.03
7	Before being marketed, dietary supplements must be tested for efficacy and safety.	F	G	4363	69.55
8	In the elderly, the use of magnesium preparations prevents muscle cramps.	F	S	1015	16.18
9	The packaging of dietary supplements must contain information on possible adverse effects resulting from their use.	F	G	3554	56.66
10	Dietary supplements are food.	T	G	4434	70.68
11	Taking dietary supplements containing calcium reduces the risk of bone fractures in the elderly.	F	S	2157	34.39
12	Dietary supplement registration requires assessing the composition of the product by the appropriate supervisory body.	F	G	3660	58.35
13	The use of antioxidants prevents the development of cancer.	F	S	1963	31.29
14	In the elderly, taking vitamin D reduces the risk of bone fractures.	F	S	1720	27.42
15	Vitamin C naturally present in food is better assimilated than synthetic.	F	S	1034	16.48
16	All dietary supplements sold in pharmacies have been tested for safety.	F	G	3785	60.34
17	Regular use of vitamin C reduces the risk of catching a cold.	F	S	2114	33.70
