# Supplementary material for: Determinants of Knowledge About Dietary Supplements Among Polish Internet Users: Nationwide Cross-sectional Study
Source: J Med Internet Res. 2021 Apr 21;23(4):e25228. doi: 10.2196/25228 (PMC8100877; doi:10.2196/25228)
Supplement: Multimedia Appendix 3 [file jmir_v23i4e25228_app3.pdf]

Supplementary File 3

The survey

The screenshots of the original survey are presented below.

Badanie naukowe – suplementy diety

Zespół naukowców z **Uniwersytetu Medycznego w Łodzi** we współpracy z **DOZ.pl** podjął się badania naukowego dotyczącego wiedzy Polaków na temat suplementów diety. W tym celu przygotowaliśmy poniższą ankietę, która składa się z 4 części, **a jej wypełnienie zajmie około 5-10 minut**.

Badanie jest całkowicie anonimowe i dobrowolne. Nie pytamy o dane osobowe. Istnieje możliwość rezygnacji z wypełniania ankiety na każdym jej etapie. Badanie zostało zaaprobowane przez Komisję Bioetyczną Uniwersytetu Medycznego w Łodzi (KE/1382/19 z dnia 15 października 2019). W razie jakichkolwiek pytań dotyczących badania można kontaktować się z dr. Michałem Karbownikiem: badanie.suplementy@gmail.com

Jeśli wyrażasz zgodę na udział w badaniu, kliknij przycisk "ROZPOCZNIJ ANKIETĘ TERAZ". Dziękujemy!

ROZPOCZNIJ ANKIETĘ TERAZ

CZĘŚĆ PIERWSZA

Co wiesz o suplementach diety?

Na następnych slajdach znajdziesz **17 stwierdzeń** na temat suplementów diety. Wskaż czy są one PRAWDZIWE czy też FAŁSZYWE. **Zaznacz odpowiedź nawet jeśli nie jesteś jej stuprocentowo pewien(-na).**

Prawidłowe odpowiedzi wraz z komentarzem eksperta poznasz po zakończeniu ankiety.

1. Przyjmowanie witaminowych i mineralnych suplementów diety zapobiega chorobom u zdrowych osób.

Wybierz jedną odpowiedź

PRAWDA

FAŁSZ

2. Jakość suplementów diety jest rutynowo badana przed dopuszczeniem ich do obrotu.

Wybierz jedną odpowiedź

PRAWDA

FAŁSZ

3. Osoby z chorobami nerek nie powinny stosować wysokich dawek witaminy C.

Wybierz jedną odpowiedź

PRAWDA | FAŁSZ

4. Stosowanie preparatów multiwitaminowych chroni przed chorobami serca.

Wybierz jedną odpowiedź

PRAWDA | FAŁSZ

5. Ta sama substancja może być sprzedawana zarówno w postaci leku, jak i suplementu diety.

Wybierz jedną odpowiedź

PRAWDA | FAŁSZ

6. Przyjmowanie nadmiernej ilości suplementów diety zawierających magnez może spowodować biegunkę i nudności.

Wybierz jedną odpowiedź

PRAWDA | FAŁSZ

7. Przed wprowadzeniem na rynek suplementy diety muszą zostać zbadane pod kątem skuteczności i bezpieczeństwa.

Wybierz jedną odpowiedź

PRAWDA | FAŁSZ

8. U osób starszych stosowanie preparatów magnezu zapobiega skurczom mięśni.

Wybierz jedną odpowiedź

PRAWDA | FAŁSZ

9. Na opakowaniach suplementów diety musi znajdować się informacja na temat możliwych skutków ubocznych wynikających z ich stosowania.

Wybierz jedną odpowiedź

PRAWDA | FAŁSZ

10. Suplementy diety to środki spożywcze.

Wybierz jedną odpowiedź

PRAWDA | FAŁSZ

11. Przyjmowanie przez osoby starsze suplementów diety zawierających wapń zmniejsza u nich ryzyko złamań kości.

Wybierz jedną odpowiedź

PRAWDA | FAŁSZ

12. Wprowadzenie suplementu diety do sprzedaży wymaga skontrolowania składu produktu przez odpowiedni organ nadzorczy.

Wybierz jedną odpowiedź

PRAWDA | FAŁSZ

13. Stosowanie antyoksydantów zapobiega rozwojowi nowotworów.

Wybierz jedną odpowiedź

PRAWDA | FAŁSZ

14. U osób starszych przyjmowanie witaminy D zmniejsza ryzyko złamań kości.

Wybierz jedną odpowiedź

PRAWDA | FAŁSZ

15. Witamina C naturalnie występująca w pożywieniu jest lepiej przyswajalna niż syntetyczna.

Wybierz jedną odpowiedź

PRAWDA

FAŁSZ

16. Wszystkie suplementy diety sprzedawane w aptekach zostały przebadane pod kątem bezpieczeństwa.

Wybierz jedną odpowiedź

PRAWDA

FAŁSZ

17. Regularne stosowanie witaminy C zmniejsza ryzyko przeziębienia.

Wybierz jedną odpowiedź

PRAWDA

FAŁSZ

CZĘŚĆ DRUGA

Reklama suplementów diety

Z reklamami suplementów diety można spotkać się w telewizji, radiu, Internecie, prasie. Czy w ciągu ostatniego tygodnia spotkałeś(-aś) się z reklamą suplementów diety?

Wybierz jedną odpowiedź

- Nie
- Tak

Chcielibyśmy poznać Twoją opinię dotyczącą **reklamy suplementów diety**. Dla każdej pary określeń zaznacz suwakiem punkt pomiędzy nimi, który jest najbliższy Twoim przekonaniom.

Informacje zawarte w reklamach suplementów diety są:

1	2	3	4	5
Nieprawdziwe (nierzetelne)				Prawdziwe (rzetelne)

1	2	3	4	5
Niewiarygodne				Wiarygodne

1	2	3	4	5
Niepełne (niekompleksowe)				Pełne (kompleksowe)

Informacje zawarte w reklamach suplementów diety są:

1 2 3 4 5

Niezrozumiałe — Zrozumiałe

1 2 3 4 5

Niejednoznaczne — Jednoznaczne

Reklamy suplementów diety:

1 2 3 4 5

Nie lubię ich — Lubię je

1 2 3 4 5

Irytują mnie — Sprawiają mi przyjemność

1 2 3 4 5

Powinno się ograniczyć ich emisję — Powinny być częściej emitowane

CZĘŚĆ TRZECIA

Pytania na temat leków

Chcielibyśmy zapytać Cię o Twoje **osobiste poglądy na temat leków**. Na kolejnych slajdach znajdują się stwierdzenia innych ludzi na temat produktów leczniczych. Prosimy, wskaż, w jakim stopniu zgadzasz się z tymi stwierdzeniami zaznaczając jedną z 5 opcji. Nie ma dobrych ani złych odpowiedzi. Jesteśmy zainteresowani Twoimi własnymi poglądami.

1. Lekarze przepisują zbyt wiele leków.

Wybierz jedną odpowiedź

- Zdecydowanie NIE zgadzam się
- Raczej NIE zgadzam się
- Nie mam zdania
- Raczej zgadzam się
- Zdecydowanie zgadzam się

2. Osoby przyjmujące leki powinny od czasu do czasu robić przerwy w ich stosowaniu.

Wybierz jedną odpowiedź

- Zdecydowanie NIE zgadzam się
- Raczej NIE zgadzam się
- Nie mam zdania
- Raczej zgadzam się
- Zdecydowanie zgadzam się

3. Większość leków uzależnia.

Wybierz jedną odpowiedź

- Zdecydowanie NIE zgadzam się
- Raczej NIE zgadzam się
- Nie mam zdania
- Raczej zgadzam się
- Zdecydowanie zgadzam się

4. Naturalne środki lecznicze są bezpieczniejsze niż leki.

Wybierz jedną odpowiedź

- Zdecydowanie NIE zgadzam się
- Raczej NIE zgadzam się
- Nie mam zdania
- Raczej zgadzam się
- Zdecydowanie zgadzam się

5. Leki powodują więcej szkody niż pożytku.

Wybierz jedną odpowiedź

- Zdecydowanie NIE zgadzam się
- Raczej NIE zgadzam się
- Nie mam zdania
- Raczej zgadzam się
- Zdecydowanie zgadzam się

6. Wszystkie leki to trucizny.

Wybierz jedną odpowiedź

- Zdecydowanie NIE zgadzam się
- Raczej NIE zgadzam się
- Nie mam zdania
- Raczej zgadzam się
- Zdecydowanie zgadzam się

7. Lekarze za bardzo polegają na lekach.

Wybierz jedną odpowiedź

- Zdecydowanie NIE zgadzam się
- Raczej NIE zgadzam się
- Nie mam zdania
- Raczej zgadzam się
- Zdecydowanie zgadzam się

8. Gdyby lekarze poświęcali więcej czasu pacjentom, przepisywaliby mniej leków.

Wybierz jedną odpowiedź

- Zdecydowanie NIE zgadzam się
- Raczej NIE zgadzam się
- Nie mam zdania
- Raczej zgadzam się
- Zdecydowanie zgadzam się

CZĘŚĆ CZWARTA

Na koniec prosimy o przekazanie nam kilku **informacji o sobie**.

Wiek (w latach)

Wybierz jedną odpowiedź

Wybierz...

Płeć

Wybierz jedną odpowiedź

- Mężczyzna
- Kobieta

Twoje wykształcenie

Wybierz jedną odpowiedź

- Podstawowe
- Średnie lub zawodowe
- Wyższe - licencjat
- Wyższe - magister
- Wyższe - doktor

Czy Twoje wykształcenie ma charakter medyczny?

Wybierz jedną odpowiedź

- Nie
- Tak

Liczba ludności w Twojej miejscowości

Wybierz jedną odpowiedź

- Poniżej 5 tysięcy
- 5-50 tysięcy
- 50-500 tysięcy
- Powyżej 500 tysięcy

Zarobki (netto na jednego członka gospodarstwa domowego na miesiąc)

Wybierz jedną odpowiedź

- Poniżej 1000 zł
- 1000 - 2000 zł
- 2000 - 3000 zł
- Powyżej 3000 zł

Jak oceniasz stan swojego zdrowia?

Wybierz jedną odpowiedź

- Słaby
- Niezły
- Dobry
- Doskonały

Oceń swoją dietę

1 2 3 4 5

Jem dużo fast foodów, chipsów, słodyczy itp.

Jem tylko zdrowe, zbilansowane posiłki

Oceń swoją aktywność fizyczną

1 2 3 4 5

Nie ćwiczę w ogóle

Intensywnie uprawiam sport 5 razy w tygodniu

Czy w ciągu ostatnich 30 dni przyjmowałeś(-aś) suplementy diety?

Wybierz jedną odpowiedź

- Nie
- Tak

Jeśli stosowałeś(-aś) ostatnio suplement diety, czy czułeś(-aś), że Ci pomaga?

Wybierz jedną odpowiedź

- Nie
- Tak
- NIE DOTYCZY - nie przyjmuję suplementów

Jeśli stosowałeś(-aś) ostatnio suplement diety, czy czułeś(-aś), że Ci szkodzi?

Wybierz jedną odpowiedź

- Nie
- Tak
- NIE DOTYCZY - nie przyjmuję suplementów

Czy obecnie palisz papierosy (tradycyjne, tytoń)?

Wybierz jedną odpowiedź

- Nie
- Nie, ale kiedyś paliłem(-am)
- Tak

Czy obecnie używasz papierosów elektronicznych?

Wybierz jedną odpowiedź

- Nie
- Nie, ale kiedyś używałem(-am)
- Tak

Czy interesujesz się tematyką suplementów diety?

1 2 3 4 5

Nie. Ten temat jest dla mnie zupełnie obojętny.

Tak! Codziennie poszukuję informacji na ten temat.

W jakim stopniu czerpiesz wiedzę na temat suplementów diety od LEKARZY?

Wybierz jedną odpowiedź

- Wcale nie
- W niewielkim stopniu
- W średnim stopniu
- W dużym stopniu

W jakim stopniu czerpiesz wiedzę na temat suplementów diety od FARMACEUTÓW?

Wybierz jedną odpowiedź

- Wcale nie
- W niewielkim stopniu
- W średnim stopniu
- W dużym stopniu

W jakim stopniu czerpiesz wiedzę na temat suplementów diety od DIETETYKÓW?

Wybierz jedną odpowiedź

- Wcale nie
- W niewielkim stopniu
- W średnim stopniu
- W dużym stopniu

W jakim stopniu czerpiesz wiedzę na temat suplementów diety od OSÓB ZNAJOMYCH (bez wykształcenia medycznego)?

Wybierz jedną odpowiedź

- Wcale nie
- W niewielkim stopniu
- W średnim stopniu
- W dużym stopniu

W jakim stopniu czerpiesz wiedzę na temat suplementów diety z MEDIÓW (czasopisma, telewizja, radio, Internet)?

Wybierz jedną odpowiedź

- Wcale nie
- W niewielkim stopniu
- W średnim stopniu
- W dużym stopniu

English language translation of the original survey (the questionnaires in the English language version are the free translations of the authors, they have not been validated for research use)

Research study – dietary supplements
Researchers from the **Medical University of Lodz** in cooperation with **DOZ.pl** undertook a research study on knowledge about dietary supplements in Poles. In order to participate you need to enter a survey, which consists of four parts and **filling it will take 5-10 minutes**. The study is fully anonymous and the participation voluntary. We ask no personal data questions. You can opt out of the survey at any stage. The study was approved by the Bioethics Committee of the Medical University of Lodz (KE/1382/19 received on 15 October 2019). In case of any questions regarding the study, you may contact dr. Michał Karbownik: badanie.suplementy@gmail.com
If you agree for participation in the study, tick a “Start the survey now” button. Thank you!

- □ Start the survey now

Part I
What do you know about dietary supplements?
In the next slides, **17 statements** about dietary supplements are following. Indicate whether a statement is TRUE or FALSE. **Tick your response even if you are not 100% sure.** Correct answers with an expert comments you will find at the end of the survey.

1. Taking vitamin and mineral supplements prevents diseases in healthy people.

Choose one answer

- ◻ TRUE
- ◻ FALSE

2. The quality of dietary supplements is routinely tested before being marketed.

Choose one answer

- ◻ TRUE
- ◻ FALSE

3. People with kidney disease should not use high doses of vitamin C.

Choose one answer

- ◻ TRUE
- ◻ FALSE

4. The use of multivitamin preparations protects against heart diseases.

Choose one answer

- ◻ TRUE
- ◻ FALSE

5. An ingredient may be sold both as a medicine or as a dietary supplement.

Choose one answer

- ◻ TRUE
- ◻ FALSE

6. Taking excessive amounts of magnesium supplements can cause diarrhea and nausea.

Choose one answer

- ◻ TRUE
- ◻ FALSE

7. Before being marketed, dietary supplements must be tested for efficacy and safety.

Choose one answer

- ◻ TRUE
- ◻ FALSE

8. In the elderly, the use of magnesium preparations prevents muscle cramps.

Choose one answer

- ◻ TRUE
- ◻ FALSE

9. The packaging of dietary supplements must contain information on possible adverse effects resulting from their use.

Choose one answer

- ◻ TRUE
- ◻ FALSE

10. Dietary supplements are food.

Choose one answer

- ◻ TRUE
- ◻ FALSE

11. Taking dietary supplements containing calcium reduces the risk of bone fractures in the elderly.

Choose one answer

- ◻ TRUE
- ◻ FALSE

12. Dietary supplement registration requires assessing the composition of the product by the appropriate supervisory body.

Choose one answer

- ◻ TRUE
- ◻ FALSE

13. The use of antioxidants prevents the development of cancer.

Choose one answer

- ◻ TRUE
- ◻ FALSE

14. In the elderly, taking vitamin D reduces the risk of bone fractures.

Choose one answer

- ◻ TRUE
- ◻ FALSE

15. Vitamin C naturally present in food is better assimilated than synthetic.

Choose one answer

- ◻ TRUE
- ◻ FALSE

16. All dietary supplements sold in pharmacies have been tested for safety.

Choose one answer

- ◻ TRUE
- ◻ FALSE

17. Regular use of vitamin C reduces the risk of catching a cold.

Choose one answer

- ◻ TRUE
- ◻ FALSE

Part II

Dietary supplements advertising

You may encounter dietary supplements ads on TV, radio, Internet, magazines. Have you had any contact with dietary supplement advertisements within the past week?

Choose one answer

- □ No
- □ Yes

We would like to know your opinion about **dietary supplements advertising**. For each pair of statements mark with a slider a point, which is the closest to your opinion.

The information conveyed by dietary supplements ads are:

untrue (unreliable)	1 – 2 – 3 – 4 – 5	true (reliable)
not trustworthy	1 – 2 – 3 – 4 – 5	credible
incomplete (incomprehensive)	1 – 2 – 3 – 4 – 5	full (comprehensive)
unclear	1 – 2 – 3 – 4 – 5	understandable
ambiguous	1 – 2 – 3 – 4 – 5	unambiguous

Dietary supplements ads:

I don’t like them	1 – 2 – 3 – 4 – 5	I like them
They annoy me	1 – 2 – 3 – 4 – 5	I enjoy them
Their emission should be limited	1 – 2 – 3 – 4 – 5	They should be broadcast more often

Part III

Questions on medicines

We would like to ask you about your **personal views about medicines in general**. In the following slides there are statements other people have made about medicines in general. Please indicate the extent to which you agree or disagree with them by ticking one of the five options. There are no right or wrong answers. We are interested in your personal views.

1. Doctors use too many medicines.

Choose one answer

- □ strongly disagree
- □ disagree
- □ uncertain
- □ agree
- □ strongly agree

2. People who take medicines should stop their treatment for a while every now and again.

Choose one answer

- □ strongly disagree
- □ disagree
- □ uncertain
- □ agree
- □ strongly agree

3. Most medicines are addictive.

Choose one answer

- ◻ strongly disagree
- ◻ disagree
- ◻ uncertain
- ◻ agree
- ◻ strongly agree

4. Natural remedies are safer than medicines.

Choose one answer

- ◻ strongly disagree
- ◻ disagree
- ◻ uncertain
- ◻ agree
- ◻ strongly agree

5. Medicines do more harm than good.

Choose one answer

- ◻ strongly disagree
- ◻ disagree
- ◻ uncertain
- ◻ agree
- ◻ strongly agree

6. All medicines are poisons.

Choose one answer

- ◻ strongly disagree
- ◻ disagree
- ◻ uncertain
- ◻ agree
- ◻ strongly agree

7. Doctors place too much trust on medicines.

Choose one answer

- ◻ strongly disagree
- ◻ disagree
- ◻ uncertain
- ◻ agree
- ◻ strongly agree

8. If doctors had more time with patients they would prescribe fewer medicines.

Choose one answer

- ◻ strongly disagree
- ◻ disagree
- ◻ uncertain
- ◻ agree
- ◻ strongly agree

PART IV

At the end please give us some **information about yourself**.

Age (in years)

Choose one answer (drop-down list)

- ◻ Below 18
- ◻ 18
- ◻ 19
- ◻ 20
- ◻ …
- ◻ 111
- ◻ 112
- ◻ 113

Sex

- ◻ Male
- ◻ Female

Your education

- ◻ Primary
- ◻ Secondary or vocational
- ◻ Higher-bachelor
- ◻ Higher-master
- ◻ Higher-doctorate

Are you medically educated?

- ◻ No
- ◻ Yes

Number of inhabitants in a place of residence

- ◻ Below 5000
- ◻ 5000-50,000
- ◻ 50,000-500,000
- ◻ Over 500,000

Earnings (monthly net household earnings per family member)

- ◻ Below 1000 PLN
- ◻ 1000-2000 PLN
- ◻ 2000-3000 PLN
- ◻ Over 3000 PLN

How do you assess your health?

- ◻ Poor
- ◻ Fair
- ◻ Good
- ◻ Excellent

Assess your diet
I eat a lot of fast food, chips, sweets, etc.   1 – 2 – 3 – 4 – 5   I only eat healthy, balanced meals

Assess you physical activity
I do no exercises at all   1 – 2 – 3 – 4 – 5   I play sport intensively 5 times a week

Have you used any dietary supplements within the past 30 days?
- □ No
- □ Yes

If you take dietary supplement, do you feel it helps you?
- □ No
- □ Yes
- □ NOT APPLICABLE – I don't use dietary supplements

If you take dietary supplement, do you feel it hurts you?
- □ No
- □ Yes
- □ NOT APPLICABLE – I don't use dietary supplements

Do you smoke conventional cigarettes?
- □ Never
- □ No, but I smoked in the past
- □ Yes

Do you use electronic cigarettes?
- □ Never
- □ No, but I used in the past
- □ Yes

Are you interested in dietary supplements issues?
No. This is completely indifferent to me.   1 – 2 – 3 – 4 – 5   Yes! Every day I look for information on this topic.

To what extent do you get knowledge about dietary supplements from PHYSICIANS?
- □ Not at all
- □ To little extent
- □ To medium extent
- □ To large extent

To what extent do you get knowledge about dietary supplements from PHARMACISTS?
- □ Not at all
- □ To little extent
- □ To medium extent
- □ To large extent

To what extent do you get knowledge about dietary supplements from DIETICIANS?

- ◻ Not at all
- ◻ To little extent
- ◻ To medium extent
- ◻ To large extent

To what extent do you get knowledge about dietary supplements from FRIENDS (with no medical education)?

- ◻ Not at all
- ◻ To little extent
- ◻ To medium extent
- ◻ To large extent

To what extent do you get knowledge about dietary supplements from MEDIA (magazines, TV, radio, Internet)?

- ◻ Not at all
- ◻ To little extent
- ◻ To medium extent
- ◻ To large extent
