# Supplementary material for: Determinants of Knowledge About Dietary Supplements Among Polish Internet Users: Nationwide Cross-sectional Study
Source: J Med Internet Res. 2021 Apr 21;23(4):e25228. doi: 10.2196/25228 (PMC8100877; doi:10.2196/25228)
Supplement: Multimedia Appendix 4 [file jmir_v23i4e25228_app4.pdf]

Supplementary File 4

Database cleaning procedure and results

Procedure

Before the analysis was performed, the data obtained from the survey was cleaned by removing the records considered potentially meaningless: these with survey completion time less than 2:30 minutes and these with more than 50% of missing values. Unrealistic age values were replaced with an indicator of a missing data. Records in which participants declared to be below 18 years of age or to have medical education (or a missing value for this variable) were removed as they were not in scope of interest of this study.

Results

The survey was displayed 24,400 times and completed 7632 times (31.28% of the displayed). The records with survey completion time less than 2:30 minutes (n=117; 1.53% of the completed) and further the records with more than 50% of missing values (n=57; 0.76% of the remaining completed) were removed to retain 7458 valid records. Unrealistic age values (more than 100 years of age) were replaced with an indicator of a missing value (n=13; 0.17% of the valid records). Data for participants who were not in scope of interest of this study, i.e. those below 18 years of age (n=47; 0.63% of the valid records) or those declaring medical education (n=1025; 13.83% of the remaining valid records) or missing this value (n=113; 1.77% of the remaining valid records) was deleted. 6273 records were present in the final database.
