# Supplementary material for: Determinants of Knowledge About Dietary Supplements Among Polish Internet Users: Nationwide Cross-sectional Study
Source: J Med Internet Res. 2021 Apr 21;23(4):e25228. doi: 10.2196/25228 (PMC8100877; doi:10.2196/25228)
Supplement: Multimedia Appendix 5 [file jmir_v23i4e25228_app5.pdf]

Supplementary File 5

Data missingness analysis

There were 1460 (0.38%) missing values in the final database of 6273 records with 61 variables each. 74 (1.18%) records presented 10% or more missing values and 566 (9.02%) records presented at least one but less than 10% of missing values. The variable with the highest number of missing values was “monthly net household earnings per family member” (n=234; 3.73%), followed by “age” (n=81; 1.29%) and "to what extent do you get knowledge about dietary supplements from media (magazines, TV, radio, Internet)” (n=79; 1.26%). Number (and frequencies) of missing values for each variable is presented in the Table below.

Table. Number and frequencies of missing values in each of the variable in the final database of 6273 records.

Variable	Missing values	
	Number	Frequency [%]
KaDS 1	14	0.22
KaDS 2	16	0.26
KaDS 3	33	0.53
KaDS 4	24	0.38
KaDS 5	20	0.32
KaDS 6	19	0.30
KaDS 7	16	0.26
KaDS 8	22	0.35
KaDS 9	18	0.29
KaDS 10	18	0.29
KaDS 11	32	0.51
KaDS 12	21	0.33
KaDS 13	27	0.43
KaDS 14	26	0.41
KaDS 15	19	0.30
KaDS 16	18	0.29
KaDS 17	22	0.35
Having contact with DS advertisements	15	0.24
BMQ 1	15	0.24
BMQ 2	26	0.41
BMQ 3	11	0.18
BMQ 4	22	0.35
BMQ 5	18	0.29
BMQ 6	21	0.33
BMQ 7	21	0.33
BMQ 8	21	0.33

Age	81	1.29
Sex	41	0.65
Education	15	0.24
Number of inhabitants	28	0.45
Earnings	234	3.73
Health	42	0.67
Use of DS	36	0.57
Positive effect of DS	42	0.67
Negative effect of DS	48	0.77
Cigarette smoking	39	0.62
Electronic cigarette use	45	0.72
Source of KaDS - medical doctors	48	0.77
Source of KaDS - pharmacists	44	0.70
Source of KaDS - dietetitians	56	0.89
Source of KaDS - friends	47	0.75
Source of KaDS - media	79	1.26

KaDS 1-17 – 1-17 item of Questionnaire on Knowledge about Dietary Supplements
DS – dietary supplements
BMQ 1-8 – 1-8 item of Beliefs about Medicines Questionnaire, General Part
Questionnaire on Trust in Advertising Dietary Supplements, responses to questions on health and diet quality as well as interest in DS were recorded in semantic differential scale with a middle value as a default – no missingness could occur in these variables

Missingness pattern was analyzed by comparing the values in each variable between observed and missing data in all the other variables. This was performed with the use of an asymptotic Mann Whitney *U* test. The results was presented in Supplementary File 6.

The analysis revealed that:

1. missingness in the initial items of the survey was mainly linked to older age and worse health
2. missingness in earnings was linked to older age, being female, being a health service user and longer survey completion time
3. missingness in the final items of the survey was linked to higher trust in advertising dietary supplements (particularly in affective and reliability domain), being a health service user and shorter survey completion time

It all provides convincing evidence against *missing completely at random* (MCAR) pattern.
