# Supplementary material for: Determinants of Knowledge About Dietary Supplements Among Polish Internet Users: Nationwide Cross-sectional Study
Source: J Med Internet Res. 2021 Apr 21;23(4):e25228. doi: 10.2196/25228 (PMC8100877; doi:10.2196/25228)
Supplement: Multimedia Appendix 7 [file jmir_v23i4e25228_app7.pdf]

Supplementary File 7

Associations of knowledge about dietary supplements predicted with the biased-corrected model with characteristics of the study participants

Table. Associations of knowledge about dietary supplements total scores predicted with the biased-corrected model with characteristics of the study participants. Estimates are expressed as β coefficient (95% confidence intervals)

Characteristics	Raw analyses[a]		Adjusted analyses[b]	
	KaDS observed	KaDS Predicted	KaDS observed	KaDS Predicted
Sociodemographic				
Age[c]	-.25 (-.28 to -.23)<br>*P*<.001	-.43 (-.45 to -.40)<br>*P*<.001	-.11 (-.13 to -.08)<br>*P*<.001	-.26 (-.28 to -.24)<br>*P*<.001
|Age-35|	-.26 (-.28 to -.24)<br>*P*<.001	-.46 (-.49 to .44)<br>*P*<.001	-.14 (-.17 to -.12)<br>*P*<.001	-.34 (-.36 to -.32)<br>*P*<.001
Sex<br>(0=female, 1=male)	.27 (.24 to .29)<br>*P*<.001	.48 (.46 to .50)<br>*P*<.001	.06 (.03 to .08)<br>*P*=.0001	.27 (.25 to .30)<br>*P*<.001
Education	.08 (.06 to .11)<br>*P*<.001	.10 (.07 to .12)<br>*P*<.001	.06 (.03 to .08)<br>*P*<.001	.06 (.04 to .08)<br>*P*<.001
Number of inhabitants	.11 (.09 to .14)<br>*P*<.001	.11 (.08 to .13)<br>*P*<.001	.06 (.04 to .08)<br>*P*<.001	.03 (.02 to .05)<br>*P*<.001
Earnings	.20 (.18 to .23)<br>*P*<.001	.28 (.26 to .31)<br>*P*<.001	.04 (.02 to .07)<br>*P*=.001	.06 (.04 to .08)<br>*P*<.001
Health-related				
Health status	.08 (.06 to .11)<br>*P*<.001	.14 (.11 to .16)<br>*P*<.001	-.01 (-.04 to .01)<br>*P*=.22	-.01 (-.03 to .00)<br>*P*=.14
Diet	-.09 (-.12 to -.07)<br>*P*<.001	-.16 (-.19 to -.14)<br>*P*<.001	.01 (-.02 to .03)<br>*P*=.53	.00 (-.02 to .02)<br>*P*=.75
Physical activity	-.04 (-.06 to -.01)<br>*P*=.005	-.06 (-.09 to -.04)<br>*P*<.001	-.02 (-.04 to .00)<br>*P*=.07	-.05 (-.06 to -.03)<br>*P*<.001
Current cigarette smoking	.01 (-.02 to .03)<br>*P*=.68	.04 (.01 to .06)<br>*P*=.002	-.03 (-.05 to -.01)<br>*P*=.01	-.01 (-.03 to .01)<br>*P*=.22
Past but not current cigarette smoking	.02 (-.00 to .04)<br>*P*=.11	.03 (.00 to .05)<br>*P*=.03	.01 (-.02 to .03)<br>*P*=.61	.01 (-.01 to .03)<br>*P*=.33
Current e-cigarette use	.09 (.06 to .11)<br>*P*<.001	.11 (.08 to .13)<br>*P*<.001	.01 (-.01 to .03)<br>*P*=.37	.00 (-.02 to .02)<br>*P*=.77
Past but not current e-cigarette use	.06 (.04 to .08)<br>*P*<.001	.08 (.05 to .10)<br>*P*<.001	.01 (-.01 to .04)<br>*P*=.26	.01 (-.01 to .02)<br>*P*=.50
Beliefs that medicines are overused	-.16 (-.19 to -.14)<br>*P*<.001	-.25 (-.27 to -.23)<br>*P*<.001	-.07 (-.09 to -.05)<br>*P*<.001	-.12 (-.14 to -.10)<br>*P*<.001
Beliefs that medicines are harmful	-.22 (-.24 to -.20)<br>*P*<.001	-.39 (-.42 to -.37)<br>*P*<.001	-.12 (-.15 to -.10)<br>*P*<.001	-.27 (-.28 to -.25)<br>*P*<.001

Dietary supplements-related				
Use of DS	-.24 (-.26 to -.22)<br>*P*<.001	-.28 (-.31 to -.26)<br>*P*<.001	-.14 (-.17 to -.12)<br>*P*<.001	-.14 (-.16 to -.12)<br>*P*<.001
Positive effect of DS	-.23 (-.26 to -.21)<br>*P*<.001	-.27 (-.29 to -.25)<br>*P*<.001	-.16 (-.18 to -.13)<br>*P*<.001	-.15 (-.17 to -.13)<br>*P*<.001
Positive effect of DS with adjustment for DS use	-.14 (-.17 to -.11)<br>*P*<.001	-.15 (-.18 to -.12)<br>*P*<.001	-.11 (-.14 to -.08)<br>*P*<.001	-.10 (-.13 to -.08)<br>*P*<.001
Negative effect of DS	.02 (-.01 to .04)<br>*P*=.18	-.01 (-.04 to .01)<br>*P*=.26	.03 (.01 to .06)<br>*P*=.003	.01 (-.01 to .03)<br>*P*=.26
Negative effect of DS with adjustment for DS use	.04 (.02 to .07)<br>*P*<.001	.01 (-.01 to .04)<br>*P*=.24	.05 (.02 to .07)<br>*P*<.001	.02 (.01 to .04)<br>*P*=.01
Having contact with DS advertisements	-.02 (-.05 to .00)<br>*P*=.11	-.04 (-.07 to -.02)<br>*P*=.001	.03 (.01 to .06)<br>*P*=.003	.03 (.01 to .05)<br>*P*<.001
Trust in advertising DS	-.48 (-.50 to -.46)<br>*P*<.001	-.85 (-.87 to -.84)<br>*P*<.001	-.37 (-.39 to -.34)<br>*P*<.001	-.73 (-.74 to -.72)<br>*P*<.001
Having contact with DS advertisements × Trust in advertising DS[d]	-.12 (-.21 to -.03)<br>*P*=.007	.00 (-.05 to .05)<br>*P*=.98	-.12 (-.21 to -.04)<br>*P*=.005	.01 (-.02 to .04)<br>*P*=.54
Interest in DS	-.24 (-.26 to -.21)<br>*P*<.001	-.35 (-.38 to -.33)<br>*P*<.001	-.10 (-.13 to -.08)<br>*P*<.001	-.17 (-.19 to -.15)<br>*P*<.001
Getting knowledge about DS from: Medical doctors[e]	-.02 (-.04 to .01)<br>*P*=.25	-.03 (-.06 to -.01)<br>*P*=.02	.01 (-.02 to .03)<br>*P*=.52	.01 (-.01 to .03)<br>*P*=.21
Getting knowledge about DS from: Pharmacists[e]	-.10 (-.13 to -.07)<br>*P*<.001	-.12 (-.14 to .09)<br>*P*<.001	-.03 (-.05 to -.00)<br>*P*=.03	-.01 (-.03 to .01)<br>*P*=.22
Getting knowledge about DS from: Dieticians[e]	.00 (-.02 to -.03)<br>*P*=.84	.01 (-.01 to .04)<br>*P*=.34	-.01 (-.03 to .02)<br>*P*=.59	-.01 (-.03 to .01)<br>*P*=.39
Getting knowledge about DS from: Friends[e]	-.14 (-.16 to -.11)<br>*P*<.001	-.26 (-.28 to -.24)<br>*P*<.001	-.13 (-.15 to -.11)<br>*P*<.001	-.26 (-.27 to -.24)<br>*P*<.001
Getting knowledge about DS from: Media[e]	-.10 (-.13 to -.08)<br>*P*<.001	-.19 (-.22 to -.17)<br>*P*<.001	-.03 (-.06 to -.01)<br>*P*=.003	-.09 (-.11 to -.07)<br>*P*<.001

Characteristics included in the predictive model (and their direct derivatives) are presented in gray.

KaDS – knowledge about dietary supplements

DS – dietary supplements

a – performed only with the variables reported

b – adjusted for: |Age-35|, Sex, Education, Number of inhabitants, Earnings, Type of online service and Calendar year – all included as linear factors

c – adjusted analyses do not include |Age-35|

d – adjusted for: Having contact with DS advertisements and Trust in advertising DS

e – the associations of KaDS with Getting KaDS from: (a particular source) were adjusted for all the other sources of KaDS
